# Using a quadruplet codon to expand the genetic code of an animal

**DOI:** 10.1093/nar/gkab1168

**Published:** 2021-12-09

**Authors:** Zhiyan Xi, Lloyd Davis, Kieran Baxter, Ailish Tynan, Angeliki Goutou, Sebastian Greiss

**Affiliations:** Centre for Discovery Brain Sciences, University of Edinburgh, UK; Centre for Discovery Brain Sciences, University of Edinburgh, UK; Centre for Discovery Brain Sciences, University of Edinburgh, UK; Centre for Discovery Brain Sciences, University of Edinburgh, UK; Centre for Discovery Brain Sciences, University of Edinburgh, UK; Centre for Discovery Brain Sciences, University of Edinburgh, UK

## Abstract

Genetic code expansion in multicellular organisms is currently limited to the use of repurposed amber stop codons. Here, we introduce a system for the use of quadruplet codons to direct incorporation of non-canonical amino acids *in vivo* in an animal, the nematode worm *Caenorhabditis elegans*. We develop hybrid pyrrolysyl tRNA variants to incorporate non-canonical amino acids in response to the quadruplet codon UAGA. We demonstrate the efficiency of the quadruplet decoding system by incorporating photocaged amino acids into two proteins widely used as genetic tools. We use photocaged lysine to express photocaged Cre recombinase for the optical control of gene expression and photocaged cysteine to express photo-activatable caspase for light inducible cell ablation. Our approach will facilitate the routine adoption of quadruplet decoding for genetic code expansion in eukaryotic cells and multicellular organisms.

## INTRODUCTION

The genetic code, universally shared among all kingdoms of life, is based on nucleotide triplet codons. Of the 64 possible triplet permutations, 61 are sense codons that map to 20 canonical amino acids and 3 are stop codons to terminate translation (1). Expanding the genetic code beyond the 20 canonical amino acids requires the reassignment of at least one codon to code for an additional amino acid. Examples in nature are the assignments of the stop codon UGA to encode selenocysteine, which is found in all three kingdoms of life, or UAG to encode pyrrolysine in methanogenic archaea and bacteria (2–5).

The development of genetic code expansion technology for the artificial expansion of the genetic code has allowed the incorporation of a wide range of synthetic non-canonical amino acids (ncAA) with properties not found in nature (6). The method involves the addition of an orthogonal aminoacyl-tRNA-synthetase (aaRS)/tRNA pair to the translational machinery of a cell. The aaRS specifically recognizes the ncAA and attaches it to the tRNA. At the ribosome, the charged tRNA then pairs with a matching codon, inserted in the reading frame of a target gene to determine the site of incorporation. Genetic code expansion has been established in a number of organisms, including single celled systems such as bacteria, yeast, mammalian cell culture, as well as multicellular organisms including *Caenorhabditis elegans*, *Drosophila*, zebrafish and mouse (7–12). The most widely used genetic code expansion system in animals is based on the pyrrolysyl-tRNA-synthetase (PylRS)/tRNA(Pyl) pair from *Methanosarcina* species. The pair is orthogonal in both prokaryotic and eukaryotic cells, which has allowed the use of directed evolution performed in *E. coli* to adapt PylRS to recognize diverse ncAA with a wide range of functionalities. Importantly, such variants evolved in *Escherichia coli* are also fully functional in eukaryotic cells. Examples of ncAA compatible with the PylRS system include various photocaged or otherwise photoresponsive amino acids, crosslinking amino acids, amino acids carrying post-translational modifications, and amino acids containing chemical handles which allow site-specific bioorthogonal labelling and attachment of functional groups (6). The codon most widely used to direct ncAA incorporation is the amber stop codon UAG, and in multicellular organisms UAG is the only codon that has thus far been used for this purpose.

Genetic code expansion depends on the availability of coding space in the form of nucleotide codons to direct incorporation, with one codon required for each ncAA to be used independently in a cell. Strategies to expand coding space include genomic recoding to free up and then reassign sense codons, which is being explored in prokaryotic cells (13,14). However, this approach is not only impractical in more complex systems, but also undesirable, as extensive genomic recoding may disrupt the normal biological function of a model system used to study biological processes. Another approach, the use of quadruplet codons does not require recoding and offers a potential expansion from 64 to 256 codons. The use of quadruplet codons has thus far however only been possible in single celled systems (15,16). The development of an efficient quadruplet decoding system in multicellular organisms would help to open a path towards further expanding the utility of ncAA mutagenesis, especially in complex systems. Apart from the prospect of allowing the concurrent use of multiple ncAA in the same cell or even the same protein, a further benefit of quadruplet codons may lie in the reduction of potential cross-decoding of endogenous triplet codons. The use of the triplet tRNA_CUA_, that recognizes the UAG stop codon, has been shown to result in incorporation of ncAA at endogenous UAG stop codons, an effect which can be reduced when employing the UAGA quadruplet codon (17,18).

Despite the advantages offered by quadruplet codons, their use has been held back due to the low ncAA incorporation efficiency compared with triplet stop codons (17,19–20). Attempts to improve quadruplet decoding have focused on the directed evolution of anticodon loops for use in bacteria and mammalian cell culture (16,18,21), and on the evolution of the ribosomal decoding centre in *E. coli* (22).

Here, we present the first method for quadruplet decoding in a multicellular organism, the nematode worm *C. elegans*. We develop hybrid tRNA variants by fusing anticodon loops optimized for quadruplet decoding (16,18,23) to tRNA scaffolds optimized for interaction with the eukaryotic translational machinery (24,25). We use our system to incorporate two distinct ncAAs, photocaged lysine (PCK) and photocaged cysteine (PCC), and show that we can achieve incorporation efficiencies that come close to those achieved using UAG triplet codons. We demonstrate the utility of the system by using quadruplet codons i) to incorporate PCK site-specifically into Cre recombinase to express a photo-activatable version of Cre which we use to optically control gene expression in live animals, and ii) to incorporate PCC into a constitutively active version of Caspase-3 to engineer a photo-activatable caspase, which we use to optically ablate *C. elegans* neurons.

This advance demonstrates the feasibility of going beyond the standard triplet-based genetic code in complex multicellular systems, and paves the way for harnessing the advantages of quadruplet decoding for genetic code expansion applications in multicellular organisms.

## MATERIALS AND METHODS

### Plasmids

All expression plasmids were constructed from pENTR plasmids using Gateway cloning (Thermo Fisher Scientific). DNA fragments were either PCR-amplified with oligonucleotide primers (Sigma-Aldrich and IDT) or ordered as gBlocks from IDT and cloned into Gateway pENTR plasmids. Synthetic genes were optimized for *C. elegans* expression (26). Expression constructs were assembled from pENTR plasmids using Gateway cloning (Thermo Fisher Scientific). All plasmids are described in Supplementary Table S3. Sequences of tRNA constructs are listed in Supplementary Table S1, sequences of protein coding genes are listed in Supplementary Table S4.

### *C. elegans* strains and maintenance

Strains were maintained under standard conditions unless otherwise indicated (27,28).

Transgenic lines were generated by biolistic bombardment into the N2, *smg-6(ok1794)* or CZ10175 genetic backgrounds using hygromycin B (Formedium) as a selection marker (29,30). Details are listed in Supplementary Table S2. Transgenic lines were maintained on hygromycin B. No hygromycin B was added to plates containing ncAA to avoid any possible physiological effects of the antibiotic on experiments, and since hygromycin B was not required to maintain transgenic arrays as animals were not grown on ncAA for more than one generation.

### Feeding of non-canonical amino acids

Photocaged lysine (PCK) and photocaged cysteine (PCC) were custom synthesized by ChiroBlock GmbH. To prepare PCK/PCC supplemented Nematode Growth Medium (NGM) agar plates, the ncAA powder was first dissolved in a small volume of 0.1 M HCl and then mixed into molten NGM agar with equimolar amounts of NaOH added to the NGM agar for neutralization (30). Worms were age synchronized by bleaching (28) and added to ncAA plates as L1 larvae. Freeze-dried OP50 (LabTIE) was reconstituted according to the manufacturer's instructions and added as food. The ncAA and ncAA plates were stored in the dark, and animals on ncAA plates were grown in the dark.

### Worm lysis and western blotting

As previously described (25), worm populations were grown to young adulthood and washed off plates using M9 buffer supplemented with 0.001% Triton-X100 to prevent animals from sticking to pipette tips. Animals were allowed to settle, the supernatant was removed and the worms were resuspended in 4× Bolt LDS sample buffer (Thermo Fisher Scientific) supplemented with 10× Bolt Reducing Agent (Thermo Fisher Scientific). The suspensions were frozen, followed by incubation in a shaking heat block at 95°C for 15 min to prepare lysates.

Samples were run on precast Bolt 4–12% Bis–Tris Plus polyacrylamide gels (Thermo Fisher Scientific) in Bolt MES SDS running buffer at 200 V for 22 min. Proteins were transferred onto a nitrocellulose membrane using an iBlot2 device (Thermo Fisher Scientific). After transfer, the membrane was blocked for 1 h at room temperature using PBST (PBS + 0.1% Tween-20) supplemented with 5% milk powder. Incubation with primary antibodies was carried out at 4°C overnight. Blots were washed 6 times for 5 min in PBST + 5% milk powder. Blots were then incubated with secondary antibodies diluted in PBST + 5% milk powder for 1 h at room temperature, followed by three washes of 5 min using PBST + 5% milk powder and one wash with PBS.

Primary antibodies used were rat anti-HA (clone 3F10, Roche) at a dilution of 1:3000 for blots exposed to film, 1:1500 for quantitative western blots, and mouse anti-GFP (clones 7.1 and 13.1, Roche) at a dilution of 1:5000 for blots exposed to film, 1:3000 for quantitative western blots. Secondary antibodies were goat anti-rat IgG(H + L)-HRP (Thermo Fisher Scientific) at a dilution of 1:5000, and horse anti-mouse IgG-HRP (Cell Signaling Technology) at a dilution of 1:5000. All dilutions were made in PBST + 5% milk powder.

Pierce ECL Western Blotting Substrate (Thermo Fisher Scientific) or SuperSignal West Femto Maximum Sensitivity Substrate (Thermo Fisher Scientific) were used as detection agents. For quantitative western blots, chemiluminescence was measured using a C-DiGit Blot Scanner (LI-COR) and band intensities analysed using ImageStudio software.

### Imaging

All imaging was performed using a Zeiss Axio Imager M2 microscope with a halogen lamp, a mercury vapor short-arc lamp and objective lens of 10x/0.3 NA air immersion, 40x/1.3 NA oil immersion and 63×/1.4 NA oil immersion. Images were processed using ZEN software (Zeiss) and ImageJ software. For imaging, animals were anaesthetized in a drop of M9 with 5 mM levamisole (Sigma-Aldrich) or 25 mM NaN_3_ (Merck) and mounted on a 3% agarose pad cast on a glass slide.

### Uncaging of PCK for Cre activation

Synchronized L1 larvae were grown for 48 h on NGM plates supplemented with 4 mM PCK or without PCK, then washed onto unseeded NGM plates and illuminated in a 365 nm CL-1000L crosslinker (UVP) at 5 mW/cm^2^ for 5 min as previously described (30). After uncaging, FUDR was added to a final concentration of 400 μM to prevent hatching of F1 progeny and thus aid scoring of animals expressing the target gene. Forty-eight hours after uncaging, animals were scored for expression of the target gene using a Leica M165FC fluorescence dissection microscope under a 2.0× objective. Each plate was independently and blindly scored by two people and the mean of both counts was used. All experiments were performed in triplicate. Significance tests were carried out using Welch's *t* test, an adaptation of Student's *t*-test for two samples with unequal variances, as a pairwise comparison between each condition at each concentration using GraphPad Prism6 software.

### Uncaging of PCC for Caspase-3 activation and apoptosis assay

To facilitate scoring and imaging, caspase expressing strains were constructed in the CZ10175 strain background, which contains a genomically integrated *mec-4p::gfp* transcriptional fusion to express GFP in the touch receptor neurons (TRNs). Synchronized L1 larvae were grown for 48h on NGM plates with either 0 or 2 mM PCC, before being washed onto unseeded 6cm NGM plates and illuminated in a CL-1000L UV crosslinker (UVP) at 365 nm, 5 mW/cm^2^ for 5 min.

To document morphological changes upon caspase activation, worms were mounted and imaged at 0, 3, 6, 12, 24, 48 h after uncaging. To quantify ablation efficiency, worms were randomly picked at 24 and 48 h after uncaging, mounted under a Zeiss Axio Imager M2 and scored for absence or presence of individual touch sensory neurons as identified using the GFP signal. Percentage values were calculated by dividing the number of missing cells by the total number of GFP positive cells expected. Each condition was scored blind and repeated in duplicate. Significance tests of the percentage of disappeared cells among all TRNs and the cell disappearance by TRN type were carried out between each condition using Fisher's exact tests for discrete distributions, by GraphPad Prism6 software.

## RESULTS

### Quadruplet decoding in *C. elegans*

Quadruplet decoding requires tRNA molecules that possess the ability to introduce +1 frameshifts during decoding, yet can function efficiently within the endogenous translational machinery which is evolutionarily optimized towards triplet codons and against translational frameshifts.

Modifications introduced into the tRNA anticodon loop outside of the anticodon can significantly improve the capability of tRNAs to introduce a +1 frameshift when delivering an amino acid to the ribosome (18). Directed evolution approaches have been used to generate improved anticodon loops, which have been used for quadruplet decoding in bacteria and cultured mammalian cells (16,18,21).

**Figure 1.**
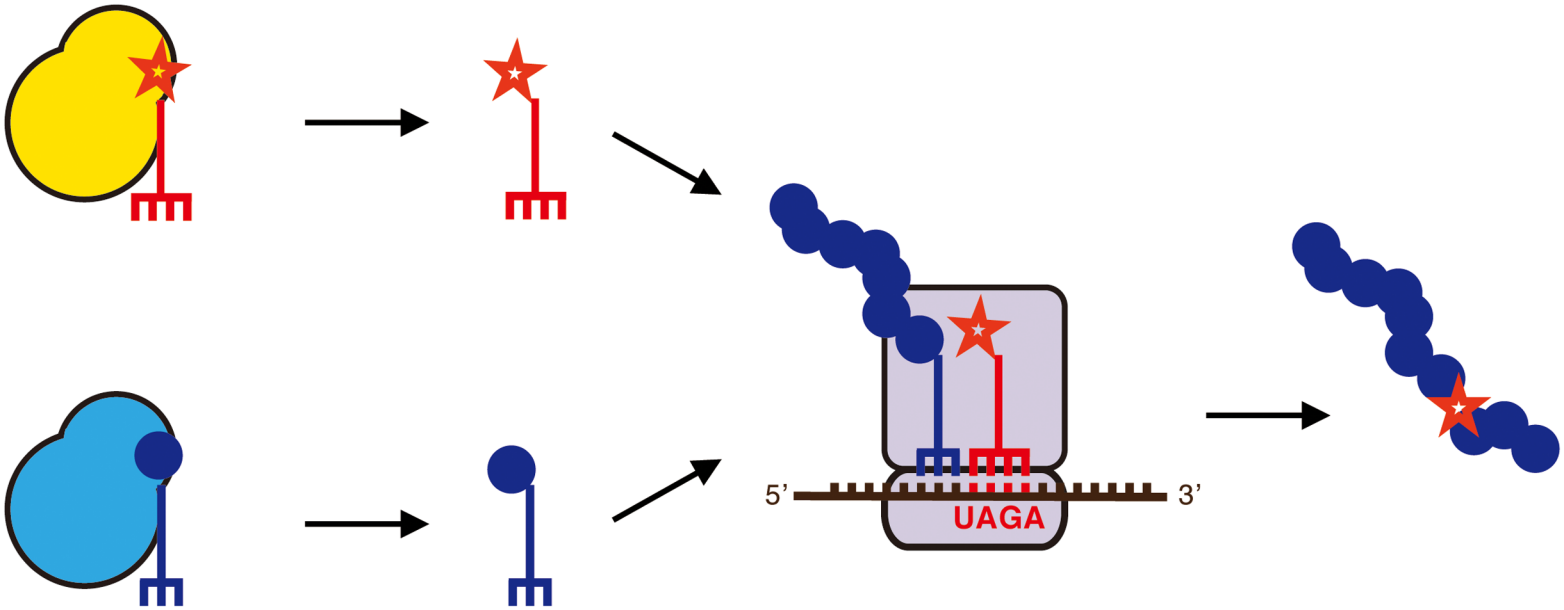
Quadruplet decoding for the incorporation of non-canonical amino acids. An orthogonal aminoacyl-tRNA synthetase (yellow) attaches a non-canonical amino acid (red star) to its cognate tRNA (red). The tRNA contains a quadruplet anticodon (red). The orthogonal components do not interact with endogenous canonical amino acids, aaRSs or tRNAs (all blue). At the ribosome, the ncAA is incorporated into the growing polypeptide chain in response to a quadruplet codon during translation.

We aimed to construct a system for quadruplet decoding in *C. elegans* based on the pyrrolysyl-tRNA synthetase (PylRS)/tRNA(Pyl) pair from *Methanosarcina* species, which is functional in *C. elegans* (10,25) and has been used for quadruplet decoding in both bacteria and cultured mammalian cells (16,18,21) (Figure 1).

To allow comparison of incorporation efficiency between a quadruplet decoding system and the established *C. elegans* UAG triplet decoding system, we decided to use the UAGA quadruplet codon, which has been used for ncAA incorporation in eukaryotic cells (23) and shares its first three nucleotides with UAG.

We first tested two tRNA(Pyl) variants, tRNA(Pyl/M7) and tRNA(Pyl/UAGA-1) which possess anticodon loops optimized for quadruplet decoding. The tRNA(Pyl/M7) contains the M7 anticodon loop and was isolated from a pool of tRNA(Pyl) variants carrying the UCCU anticodon that decodes AGGA (16). The UAGA-1 anticodon loop was isolated from a pool of tRNA(Pyl) variants in a screen for improved UAGA decoding (18,23). Interestingly, the anticodon loop of tRNA(Pyl/M7) is similar to that of tRNA(Pyl/UAGA-1) and compared to the wild type tRNA(Pyl)_CUA_, M7 and UAGA-1 share three mutations in close proximity to the anticodon (Supplementary Table S1, Supplementary Figure S1). We therefore surmised that the M7 variant might be effective even when the anticodon is changed from UCCU to UCUA for decoding UAGA.

**Figure 2.**
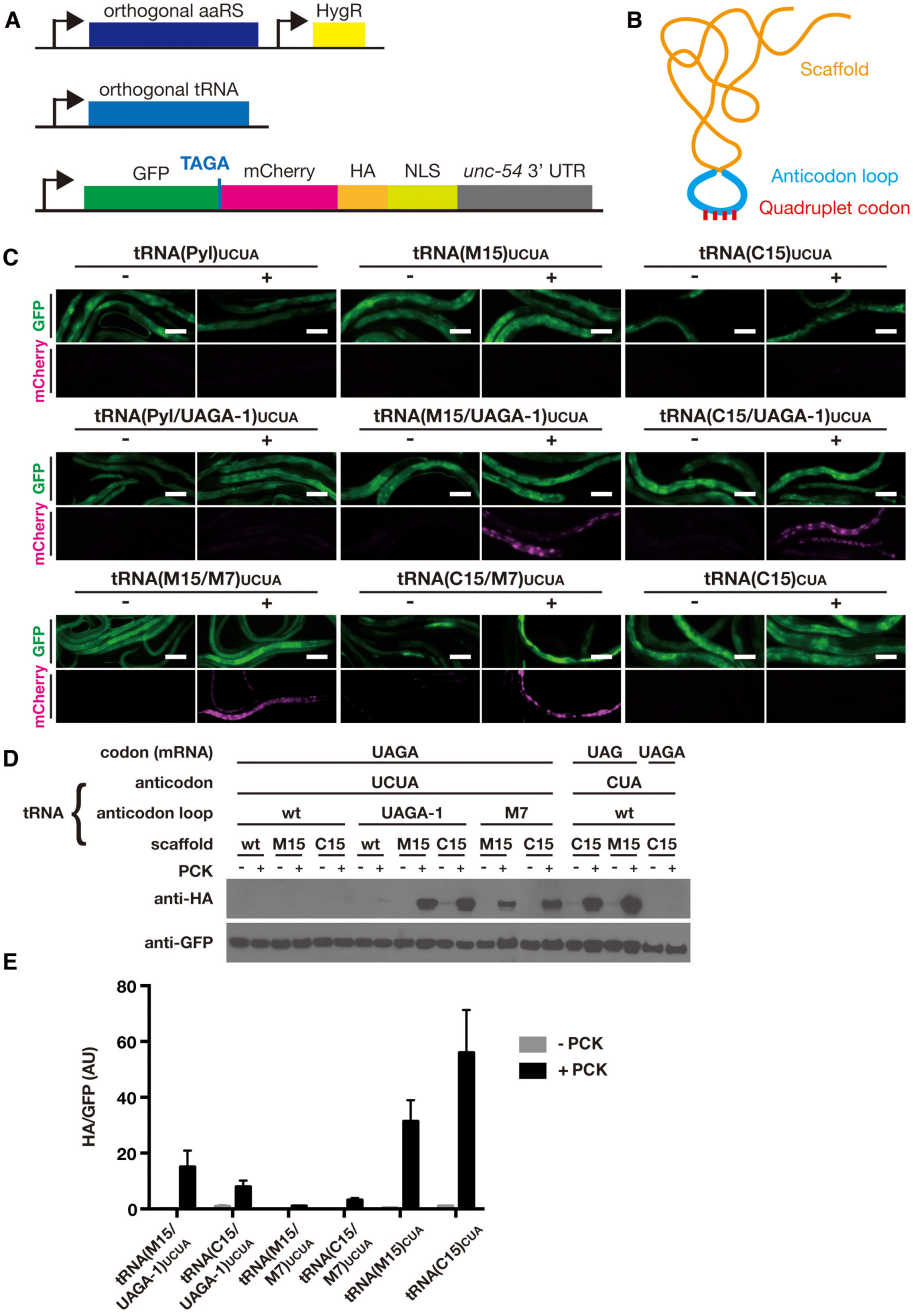
Efficient quadruplet decoding using hybrid tRNAs. (**A**) Genetic constructs for the quadruplet decoding machinery consisting of aaRS and orthogonal tRNA. The machinery is co-expressed with a fluorescent reporter for ncAA incorporation, using either a TAG or TAGA codon as specified in the figure and the legend below. (**B**) Schematic of hybrid tRNA consisting of scaffold and anticodon loop. (**C**) Fluorescence microscopy images of randomly selected worms expressing different tRNA variants together with the aaRS PCKRS and the fluorescent reporter containing a TAGA codon to detect quadruplet decoding. Animals were grown in the presence (‘+’) or absence (‘–’) of 1 mM PCK. ‘Pyl’, ‘M15’ and ‘C15’ denote scaffolds, ‘UAGA-1’ and ‘M7’ denote anticodon loops. For tRNAs containing wild type anticodon loops only the scaffold type is indicated. Scale bar = 100 μm. (**D**) Western blots of lysates made from transgenic lines shown in (C). Full-length reporter protein was detected using anti-HA antibody, samples were normalized using anti-GFP antibody. (**E**) Quantitative western blots of the constructs showing the highest levels of incorporation in (C) and (D). Error bars show the standard error of the mean. For all constructs, two independent lines were quantified.

To test the functionality of the quadruplet decoding tRNAs, we constructed transgenic *C. elegans* lines carrying three plasmids (Figure 2A): (i) a plasmid expressing the PylRS variant NES::PCKRS that recognizes photocaged lysine (PCK) (25,31) and at its N-terminus contains a nuclear export signal (NES) derived from human Smad4 (32), which dramatically improves the efficiency of the enzyme (25); (ii) tRNA(Pyl/M7)_UCUA_ or tRNA(Pyl/UAGA-1)_UCUA_ with expression driven by the ubiquitous RNA polymerase III promoter *rpr-1p* (33) and (iii) a fluorescent reporter for PCK incorporation. The fluorescent reporter consists of a GFP gene separated by a UAGA codon from a downstream mCherry gene, followed by an HA tag and a nuclear localization sequence (NLS) (Figure 2A). Incorporation at the UAGA codon thus results in production of a GFP::mCherry fusion protein that localizes to the cell nucleus due to the C-terminal NLS. Expression of the protein coding genes was driven by the ubiquitous *sur-5p* promoter (34). To construct strains, we used a genetic background containing a deletion in the *smg-6* gene, knocking out the nonsense mediated decay machinery (35). This dysfunction in nonsense mediated decay helps to stabilize the reporter mRNA. All transgenic strains were generated by biolistic bombardment, which can yield strains carrying the transgenes in extrachromosomal arrays of varying copy number or, at low frequency, strains with genomically integrated transgenes (29). All strains we generated contained the transgenes in the form of extrachromosomal arrays.

To assess PCK incorporation, we grew transgenic animals for 48 h on Nematode Growth Medium (NGM) agar plates supplemented with 1 mM PCK. We observed very weak, barely visible mCherry fluorescence indicative of PCK incorporation in a minority of animals when using tRNA(Pyl/UAGA-1)_UCUA_, (Figure 2C, Supplementary Figure S2A–C). We confirmed presence of the full-length GFP::mCherry::HA product following incorporation by western blot using an anti-HA antibody to detect the C-terminal HA tag, albeit a band was only visible after extended exposure of the blot to film (Figure 2D, Supplementary Figure S3). As expected, no incorporation was detected in the absence of PCK. In contrast to tRNA(Pyl/UAGA-1)_UCUA_, we observed no incorporation when using tRNA(Pyl)_UCUA_, which contains the UCUA anticodon in a wild type anticodon loop (Figure 2C, D, Supplementary Figures S2, S3). We could not find any strains showing detectable incorporation when using tRNA(Pyl/M7)_UCUA_.

### Optimized hybrid tRNAs for efficient quadruplet decoding

The archeal tRNA(Pyl) contains sequence elements that differ from canonical mammalian tRNAs and these structural differences disfavour the interaction of tRNA(Pyl) with the eukaryotic translational machinery (24). The efficiency of ncAA incorporation in mammalian cells and in *C. elegans* can be improved many-fold by the introduction of facilitative structural elements into the tRNA scaffold (24,25). As these optimizing tRNA mutations are outside the anticodon loop, we set out to assess the possibility of constructing hybrid tRNAs for quadruplet decoding by combining optimized anticodon loops with scaffolds optimized for the eukaryotic translational context (Figure 2B).

We based the new hybrid tRNAs on two optimized scaffolds originally engineered for usage in mammalian cells, M15 and C15, which drastically increase ncAA incorporation efficiency at UAG triplet codons in *C. elegans* (25). We combined the M15 and C15 scaffolds with the M7 and UAGA-1 anticodon loops to create four hybrid tRNA variants: tRNA(M15/M7)_UCUA_, tRNA(C15/M7)_UCUA_, tRNA(M15/UAGA-1)_UCUA_ and tRNA(C15/UAGA-1)_UCUA_ (Supplementary Table S1, Supplementary Figure S1). All four hybrid variants contained the UCUA anticodon to decode the UAGA quadruplet codon. We generated transgenic strains co-expressing the hybrid tRNA variants together with the synthetase NES::PCKRS and the GFP::UAGA::mCherry reporter (Figure 2A).

We then tested incorporation efficiencies by growing transgenic animals on NGM plates supplemented with 1 mM PCK. In the strains expressing hybrid tRNAs, we saw a striking improvement in incorporation efficiency as compared to the strains expressing tRNAs with only the anticodon loops optimized (Figure 2C, D). The UAGA-1 anticodon loop appeared to outperform the M7 anticodon loop, while both the M15 and the C15 scaffolds showed comparable improvements to incorporation efficiency. The improvement in incorporation rates was due to the combined effect of optimized scaffold and loops, since tRNA(M15)_UCUA_ and tRNA(C15)_UCUA_, which combine an optimized scaffold with the wild type anticodon loop showed no incorporation or only barely detectable incorporation (Figure 2C,D).

We performed quantitative western blots comparing incorporation rates of the four hybrid quadruplet tRNAs against triplet codon incorporation rates achieved using the parent tRNA molecules tRNA(M15)_CUA_ and tRNA(C15)_CUA_ incorporating at a UAG triplet codon. While we observed the highest incorporation rates when using the triplet incorporation system, the best performing quadruplet tRNA(M15/UAGA-1) reached 30–50% of the levels of incorporation observed for the triplet tRNAs M15 and C15 (Figure 2E). Strikingly, the best performing quadruplet tRNA(M15/UAGA-1) showed a 4-fold improvement of incorporation compared to the standard, non-optimized UAG triplet system consisting of wild type tRNA(Pyl)_CUA_ and PCKRS without an N-terminal nuclear export sequence (Supplementary Figure S4). We found incorporation rates of up to 2.5% in the best quadruplet tRNA strain, as compared to 6% for the best triplet tRNA strain (Supplementary Figure S5). Due to the high incorporation efficiency of the improved quadruplet system, we decided to perform all further experiments in the wild type N2 genetic background with functional nonsense mediated decay.

### Application of quadruplet decoding for the expression of photocaged Cre recombinase and optical control of gene expression in *C. elegans*

**Figure 3.**
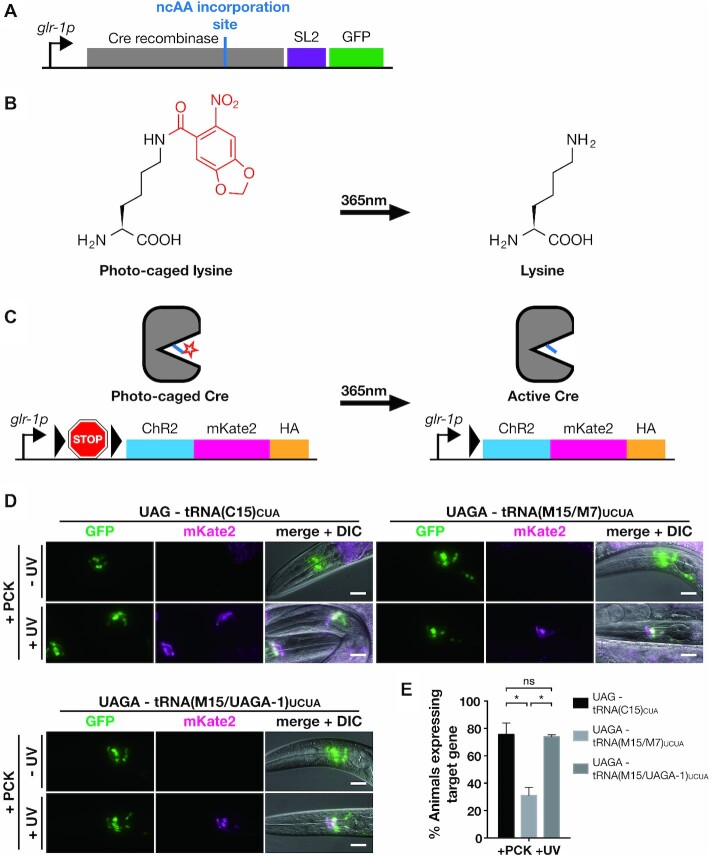
Using quadruplet codons to express photocaged Cre recombinase. (**A**) Genetic constructs for expression of photocaged Cre. Photocaged lysine (PCK) replaces the active site lysine K201 of Cre. The incorporation site is specified using either a TAG or TAGA codon. Photocaged Cre is co-expressed in an operon together with GFP. (**B**) 6-Nitropiperonyl-l-lysine, ‘photocaged lysine’. The caging group is stable in visible light and can be removed by brief illumination with 365 nm light. (**C**) Optical activation of Cre. When introduced in place of K201, the caging group of PCK (red star) blocks Cre activity. Transcription of the target gene is blocked by a transcriptional terminator (‘STOP’) inserted between the gene and its promoter. The terminator is flanked by loxP sites (black triangles). Uncaging activates Cre, which removes the transcriptional terminator, thereby activating target gene expression. (**D**) Fluorescence microscopy images of transgenic lines expressing the PCK incorporation machinery utilizing either triplet (‘UAG’) or quadruplet (‘UAGA’) codons to direct incorporation at K201. Animals were grown in the presence of 4 mM PCK. Scale bars = 20 μm. (**E**) Quantification of photocaged Cre activation for the lines depicted in (D). Animals were grown in the presence of 4 mM PCK for 48 h. After uncaging, 30 animals were scored for expression of the target gene. The mean of three independent experiments is shown. Error bars show the standard error of the mean. Significance is derived from P values of Welch's t test.

We next decided to assess the utility of the optimized quadruplet-decoding system by using it to express a light controllable version of Cre recombinase. Lysine residue K201 in the catalytic site of Cre recombinase is required for activity and replacing it with PCK renders the enzyme inactive. Uncaging by short illumination with 365 nm light restores Cre activity and can thus be used to activate Cre and switch on expression of Cre target genes (36) (Figure 3A–C, Supplementary Figure S6). We have previously established photocaged Cre as a tool for optical control of gene expression in *C. elegans* (25), using a UAG triplet codon system to direct incorporation of PCK. Here, instead of UAG, we used the UAGA quadruplet codon to direct incorporation of PCK in place of the active site lysine K201. To decode UAGA, we tested the two quadruplet tRNA variants based on the M15 scaffold, tRNA(M15/M7)_UCUA_ and tRNA(M15/UAGA-1)_UCUA_.

We generated transgenic strains expressing the quadruplet-decoding system together with a construct expressing photocaged Cre, containing TAGA in the place of the codon for lysine K201 and a reporter construct to monitor Cre activity that we have previously described (25). The reporter construct consists of a channelrhodopsin gene fused to red fluorescent mKate2, separated from its promoter by a transcriptional terminator sequence flanked by loxP sites (Figure 3C). Cre activates transcription of the reporter and results in red fluorescence. To visualize presence of the construct encoding photocaged Cre, we expressed it as part of an artificial operon together with the marker GFP (Figure 3A). All protein-coding genes were expressed from the *glr-1p* promoter that is active in *C. elegans* glutamatergic neurons (25,37).

We grew age-synchronized populations of transgenic worms on NGM agar plates supplemented with 4 mM PCK for 48h, from the L1 larval stage. We then activated photocaged Cre by illumination with 365 nm light and scored for expression of the target gene 24h after activation. For both quadruplet tRNA variants, we observed animals that showed clear expression of ChR2::mKate2 in neurons expressing photocaged Cre. In contrast, we saw no ChR2::mKate2 in animals grown on PCK but not subjected to UV illumination (Figure 3D). We quantified Cre activation frequency as a measure of the production of photocaged Cre and thus PCK incorporation for the best performing strain for each tRNA variant. We found that the most efficient quadruplet tRNA variant tRNA(M15/UAGA-1)_UCUA_ performed equally well as our best triplet strain which contains the triplet variant tRNA(C15)_CUA_, with over 70% of animals showing expression of the target gene ChR2::mKate2. The less efficient tRNA(M15/M7)_UCUA_ still showed optically triggered Cre activity in 30% of animals (Figure 3E). The optimized quadruplet incorporation machinery thus performs with a level of efficiency close to an optimized UAG triplet decoding system.

### Quadruplet directed incorporation of photocaged cysteine

In *C. elegans* the only photocaged amino acids that have been successfully incorporated into proteins are photocaged lysine and photocaged tyrosine (25,38). We decided to apply quadruplet decoding to a further ncAA, namely a photocaged cysteine (PCC), thus expanding the repertoire of photocaged amino acids for *C. elegans*. Cysteine is an attractive target for photo-caging, since key cysteine residues are present in many biologically important protein classes, including ubiquitin ligases, phosphatases, and proteases such as deubiquitinases and caspases.

**Figure 4.**
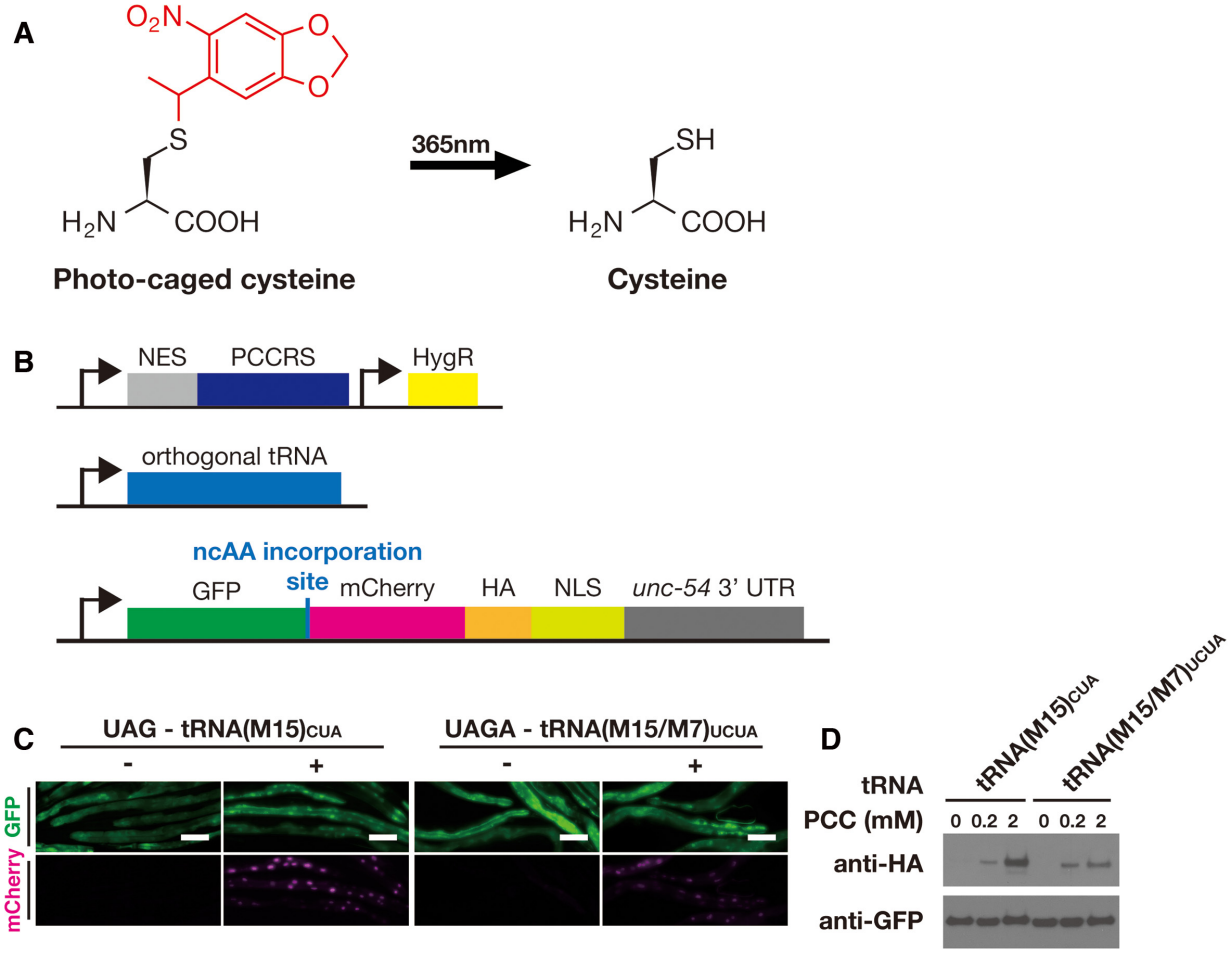
Incorporation of photocaged cysteine in *C. elegans*. (**A**) The nitropiperonyl caging group (red) of photocaged cysteine (PCC) can be removed by 365 nm illumination. (**B**) Genetic constructs for the machinery to incorporate PCC, consisting of the PCC specific aaRS PCCRS with a nuclear export sequence attached to the N-terminus (NES::PCCRS), orthogonal tRNA with either a triplet CUA or quadruplet UCUA anticodon, and the fluorescent reporter GFP::mCherry::HA::NLS. (**C**) Fluorescence microscopy images of transgenic lines expressing either the triplet tRNA(M15)_CUA_ or the quadruplet tRNA(M15/M7)_UCUA_, together with NES::PCCRS and the fluorescent reporter containing either a triplet (‘UAG’) or quadruplet (‘UAGA’) codon at the ncAA incorporation site between GFP and mCherry as shown in (B). Animals were grown in the presence (‘+’) or absence (‘–’) of 2 mM PCC. Scale bar 100 μm. (**D**) Western blots of the lines shown in (C) and grown in the absence of PCC or the presence of either 0.2 mM or 2 mM PCC. Full-length reporter protein was detected using anti-HA antibody, samples were normalized using anti-GFP antibody.

We used cysteine caged with a nitropiperonyl group (Figure 4A, Supplementary Figure S7), which has an absorption maximum close to 365 nm and is therefore more amenable to *in vivo* use than the alternative *ortho*-nitrobenzyl caging group whose optical removal can be challenging (39). Nitropiperonyl PCC is recognized by PCCRS, a variant of PylRS and has previously been established in mammalian cultured cells. We introduced the previously described PCCRS mutations (39) into a PylRS gene from *Methanosarcina mazei*, optimized for expression in *C. elegans*. To improve incorporation efficiency, we again added the Smad4 nuclear export sequence to the N-terminus to generate NES::PCCRS.

We created transgenic strains expressing NES::PCCRS together with either tRNA(M15)_CUA_ or tRNA(M15/M7)_UCUA_. To visualize incorporation, we used our fluorescent GFP::mCherry reporter, with the two fluorescent proteins separated either by a UAG triplet codon to assay incorporation by the triplet tRNA(M15)_CUA_, or separated by a UAGA quadruplet codon to assay incorporation by the quadruplet tRNA(M15/M7)_UCUA_ (Figure 4B).

When we grew transgenic animals on NGM agar plates supplemented with PCC, we observed strong red fluorescence appearing within 24 h for both the triplet and the quadruplet system (Figure 4C, Supplementary Figure S8). We further confirmed the identity of the full-length reporter protein by western blot against the C-terminal HA tag (Figure 4D). We thus show that PCC can be efficiently incorporated in *C. elegans* using either a UAG triplet or a UAGA quadruplet codon.

### Quadruplet codon directed incorporation of photocaged cysteine into Caspase-3 for optical control of apoptosis induction in *C. elegans*

We then decided to use the quadruplet system to incorporate PCC into the active site of human Caspase-3, a caspase that is routinely used for genetically controlled cell ablation in *C. elegans* (40). By introducing PCC we aimed to optically control activity of the caspase and thus use light to ablate cells.

As a key executor of the apoptotic pathway, wild type Caspase-3 is synthesized as an inactive zymogen with its subunits arranged in such a way as to be sterically prevented from folding into the active conformation. The zymogen is converted into the active caspase by cleavage at internal proteolytic sites and subsequent rearrangement of its long and short subunits to form the active enzyme (Supplementary Figure S9A) (41,42). By switching the order of the subunits on the polypeptide chain, it is possible to express a constitutively active form of Caspase-3 that does not require proteolytic cleavage for activation (Supplementary Figure S9B) (41).

**Figure 5.**
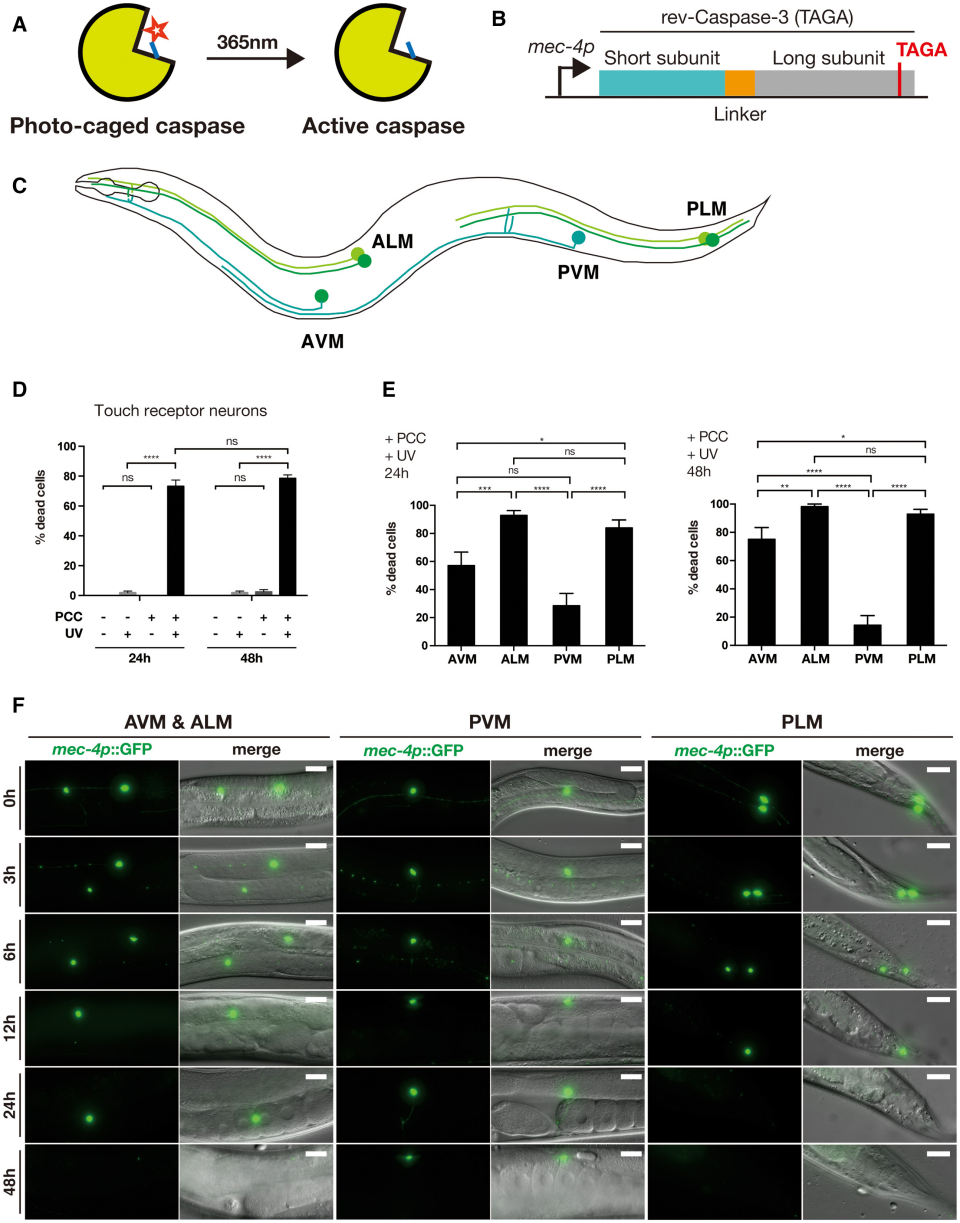
Optical control of caspase activity using quadruplet codon directed incorporation of photocaged cysteine. (**A**) Photocaged caspase with PCC (red star) in its active site is inactive, until it is uncaged by 365 nm light. (**B**) Genetic construct for expression of photocaged Caspase-3. Photocaged cysteine is inserted in place of the catalytic cysteine using a TAGA quadruplet codon. (**C**) Schematic of the six *C. elegans* touch receptor neurons (TRN). AVM and PVM are single cells, while ALM and PLM are bilaterally symmetric neurons pairs. (**D**) Percentages of TRN killed in animals grown in the presence (‘+PCC’) or absence (‘−PCC’) of photocaged cysteine and either uncaged by illumination with 365 nm (‘+UV’) or kept in the dark (‘−UV’). A minimum of 25 animals were randomly picked for each treatment group and scored for missing neurons either 24 or 48 h after the uncaging step. The average for each group is shown. Error bars represent the standard error of the mean. Significance between groups was determined by Fisher's exact tests. (**E**) Percentage of disappeared cells by TRN type of the ‘+PCC +UV’ animals 24 h (left graph) and 48 h (right graph) post UV uncaging. Error bars represent the standard error of the mean. Significance was determined by Fisher's exact tests. (**F**) Fluorescent images of neurons 0, 3, 6, 12, 24, 48 h after UV uncaging. GFP signals were superimposed onto DIC images to generate merged images. Scale bars = 20 μm.

We based our design of photocaged caspase on the constitutively active form of Caspase-3, replacing the catalytically critical cysteine residue in the caspase active site with PCC. In this arrangement, activity of the enzyme would be blocked by the caging group on PCC and activity could later be restored by removing the caging group via 365 nm illumination (Figure 5A). The activation of photocaged caspase should then result in the death and removal of the targeted cell.

We generated transgenic strains expressing the quadruplet incorporation machinery for PCC, consisting of NES::PCCRS and tRNA(M15/M7)_UCUA_ together with a construct encoding for photocaged caspase, which contained a TAGA quadruplet codon in place of the catalytic cysteine (Figure 5B). We targeted expression of NES::PCCRS and photocaged caspase to the *C. elegans* touch receptor neurons (TRN) using the *mec-4p* promoter (Figure 5B, C) (43). We chose the TRNs, a neuron class consisting of six cells, since they are easy to distinguish and score under a microscope. We generated transgenic lines in the CZ10175 strain, which carries a genomically integrated transcriptional *mec-4p*::*gfp* fusion, labelling the TRN neurons, but is otherwise wild type. The GFP fluorescence allowed us to easily score for presence or absence of neurons to quantify removal of cells upon photocaged caspase activation.

We grew age-synchronized populations of transgenic worms on NGM agar plates supplemented with 2 mM PCC from the L1 larval stage for 3 days until the late L4 larval stage. To activate photocaged caspase, we then uncaged PCC using 365 nm illumination and scored the number of neurons 24 h after uncaging. We found that animals grown on PCC and treated with UV had lost an average of 73% of TRNs (Figure 5D). This number did not increase significantly after 48h, indicating that the majority of cell removal due to the optically induced caspase activity occurred within 24 h after activation.

Interestingly, we found that among the TRNs some cell types were removed more effectively than others, with the ALM and the PLM neuron pairs showing the greatest proclivity for photocaged caspase induced cell ablation. 48h post activation, almost 100% of ALM and PLM cells had disappeared. This was in contrast to the AVM neuron (75% disappeared after 48h) and especially the PVM neuron (14% disappeared) (Figure 5E).

When we examined cells at different time points after caspase activation, we observed changes in cell morphology consisting of fragmentation of cell processes as a first sign of initiated cell degeneration, followed by the cells taking on a spherical appearance and finally complete disappearance (Figure 5F).

We could therefore demonstrate that the improved quadruplet incorporation machinery allows for the use of PCC to produce sufficient photocaged caspase for optically induced caspase dependent cell ablation in *C. elegans* neurons.

## DISCUSSION

The site-specific incorporation of ncAA in multicellular organisms was until now limited to the use of reassigned UAG stop codons. We report here the first instance of genetic code expansion using quadruplet decoding in an animal. Our system reaches incorporation efficiencies for a quadruplet codon approaching those observed with optimized UAG triplet decoding tRNAs and surpassing those of a non-optimized UAG triplet system. We achieve this by establishing the use of hybrid tRNA variants, which combine scaffolds optimized for use in eukaryotic cells and anticodon loops evolved for decoding of quadruplet codons.

We observed striking efficiency improvements for all four tested hybrid tRNAs compared to their parent molecule scaffolds and anticodon loops. Importantly, this suggests that it may be possible to treat anticodon loops and scaffolds as independent biological parts that can be combined to allow the creation of novel tRNA hybrids. An immediate benefit would be the possibility of independently evolving such parts before easily recombining the newly evolved structural features into tRNA hybrids. This is an attractive prospect, given that several mutually orthogonal and efficient aminoacyl-tRNA-synthetase/tRNA pairs based on the PylRS/tRNA(Pyl) system have recently been developed for bacteria and mammalian cells (44,45), albeit they have not yet been established in animals. In order to utilize such mutually orthogonal pairs, it will be necessary to have access to sufficient coding space to allow for the possibility of incorporating more than one and up to several ncAAs independently within the same cell. Since the identity elements of these newly developed orthogonal PylRS/tRNA(Pyl) pairs lie within the scaffold region and outside the anticodon loop it should be possible to use them for the construction of new orthogonal quadruplet hybrid tRNAs and thus facilitate the expansion of the coding space in cells and organisms. Likewise, it should be possible to evolve or rationally redesign scaffolds using a UAG triplet anticodon and then combine such improvements with independently evolved specialized quadruplet anticodon loops.

Importantly, we were also able to show that improvements in anticodon loops resulting in increased quadruplet decoding efficiency are to some extent independent of the anticodon itself. We found that the M7 anticodon loop with a UCUA anticodon could efficiently decode the UAGA quadruplet, even though the M7 anticodon loop was originally evolved for the UCCU anticodon and decoding of the quadruplet AGGA (16). This suggests the possibility of directly utilizing the hybrid tRNAs presented here for decoding other quadruplet codons without the need for further optimization, by simply replacing the anticodon.

We show that it is possible to achieve quadruplet incorporation efficiencies that, while still below those observed with an optimized UAG triplet system, can easily produce sufficient amounts of protein for *in vivo* applications. Indeed, we find that in a direct comparison using photocaged Cre recombinase, our quadruplet system performs as efficiently as an optimized UAG triplet system.

We were able to use quadruplet decoding in conjunction with photocaged cysteine to express photocaged caspase as a novel tool for cell ablation in *C. elegans*. Cell ablation is a widely used approach to uncover the function of cells within the organismal context. A number of cell-ablation methods have been developed in *C. elegans* and other systems, namely laser ablation (46), expression of toxic dominant mutations in ion channels such as *mec-4(d)* (47) or *trp-4(d)* (48) and optogenetic approaches using singlet oxygen generators such as MiniSOG (49). Laser ablation, while allowing the targeting of individual cells, can be technically challenging, and is in most cases not possible to perform without affecting surrounding tissues in adult worms with a fully developed nervous system. The expression of toxic proteins is dependent on the availability of cell specific promoters and does not offer temporal precision. Singlet oxygen generators may require prolonged illumination and are not compatible with imaging approaches utilizing the same visible wavelengths used to activate singlet oxygen production. The use of an optically controlled caspase allows temporally controlled removal of cells, while the use of a UV responsive photocage allows for the possibility of using visible wavelengths at the same time, for example for imaging or the control of additional optogenetic tools. A further, general, advantage of genetic code expansion based methods is that production of the photocaged protein is controlled through the availability of ncAA, making them inherently inducible systems. Animals therefore only need to be kept away from wavelengths capable of uncaging when grown in the presence of ncAA. Furthermore, short, low-powered UV pulses delivered using a microscope-mounted laser may allow the targeted removal of individual cells without affecting surrounding tissues. We have previously demonstrated the feasibility of targeted laser uncaging in *C. elegans* for the activation of photocaged Cre recombinase (25).

The use of quadruplet codons allows the concurrent incorporation of more than one ncAA (21,22) and our work will make it possible to explore the use of quadruplets for this purpose in animals and other multicellular organisms. Furthermore, the repurposing of triplet stop codons as sense codons for genetic code expansion is accompanied by the possible misincorporation of ncAA at endogenous stop codons (50). The use of the UCUA anticodon, which decodes UAGA, was previously shown to result in significantly lower levels of cross-decoding and thus misincorporation at UAGN codons other than UAGA (18). This finding suggests the interesting possibility of using a quadruplet codon could be advantageous even for the incorporation of a single ncAA as it might reduce such misincorporation events, which may be especially undesirable for experimental approaches such as protein labelling or crosslinking, where it may contribute to non-specific background signals. Our improved quadruplet decoding system should allow the routine use of quadruplet codons and thus the exploration of this possibility. In fact, since our system outperforms the standard wild type PylRS/tRNA(Pyl)_CUA_ triplet pair that is currently used for most genetic code expansion work in animals and cultured cells, a switch to quadruplets will not come at the price of reduced efficiency for most labs.

In summary, we have succeeded in adding quadruplet codons to the genetic coding repertoire of a multicellular organism, by introducing the use of hybrid tRNAs combining optimized anticodon loops and scaffolds. The system we have developed reaches efficiency levels for the site-specific incorporation of a ncAA that come close to those observed for UAG triplet codons. Using photocaged amino acids encoded by quadruplet codons, we were able to express photocaged Cre recombinase and photocaged caspase allowing us to optically control gene expression and to induce cell death in *C. elegans* neurons. We therefore demonstrate that quadruplet codons are a viable alternative to triplet codons for genetic code expansion. We expect that the improvements we have demonstrated will be applicable in single- and multicellular eukaryotic systems beyond *C. elegans*.

## DATA AVAILABILITY

All data are included in the manuscript and the supplementary file. Sequences that have been used for transgenic constructs are listed in the supplementary file.

## Supplementary Material

gkab1168_Supplemental_File
