# Supplementary material for: Using a quadruplet codon to expand the genetic code of an animal
Source: Nucleic Acids Res. 2021 Dec 9;50(9):4801–12. doi: 10.1093/nar/gkab1168 (PMC9122531; doi:10.1093/nar/gkab1168)
Supplement: gkab1168_Supplemental_File [file gkab1168_supplemental_file.pdf]

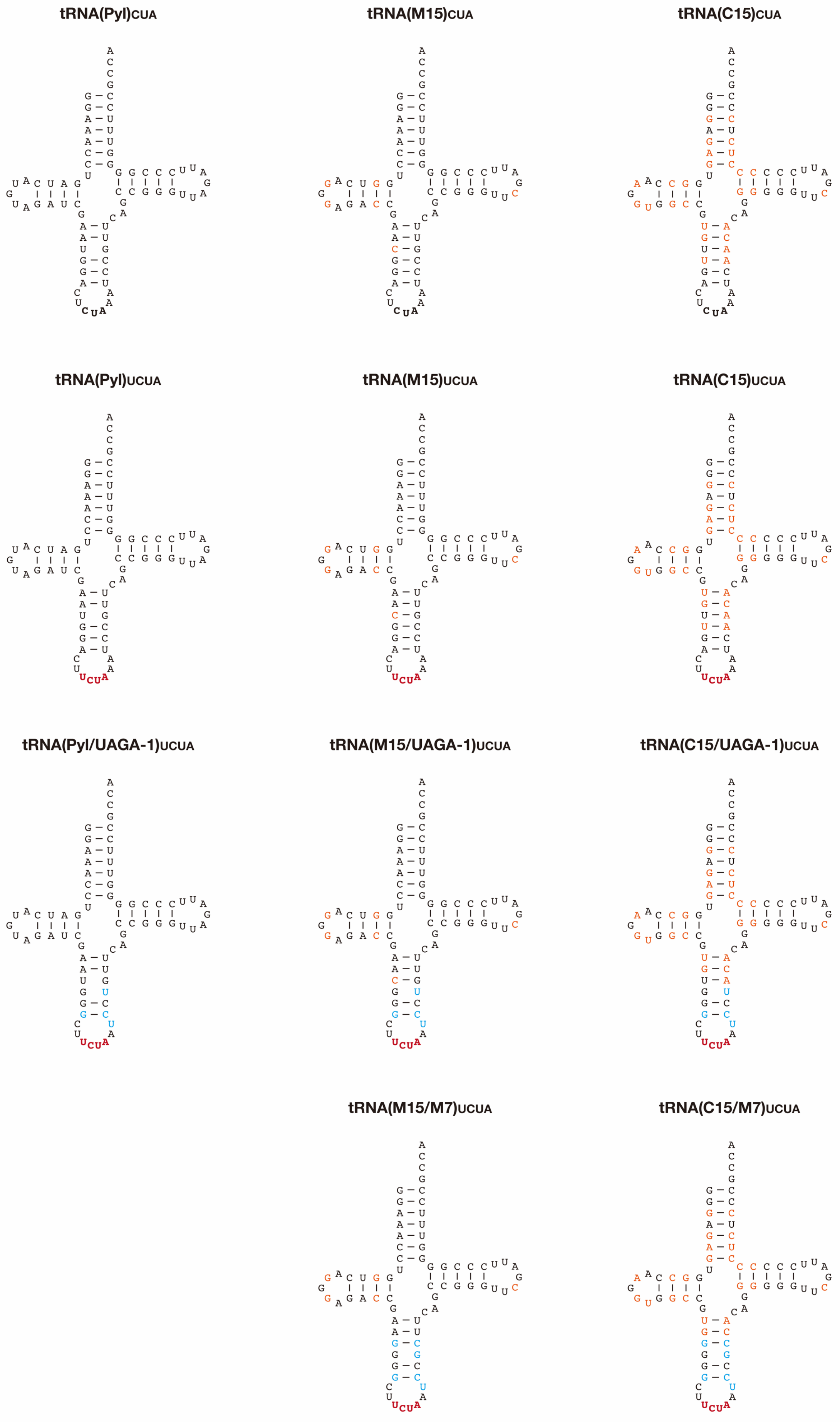
tRNA(Pyl)CUA
tRNA(M15)CUA
tRNA(C15)CUA
tRNA(Pyl)UCUA
tRNA(M15)UCUA
tRNA(C15)UCUA
tRNA(Pyl/UAGA-1)UCUA
tRNA(M15/UAGA-1)UCUA
tRNA(C15/UAGA-1)UCUA
tRNA(M15/M7)UCUA
tRNA(C15/M7)UCUA

Supplementary Figure 1: Schematics of wild type tRNA(Pyl)$_{CUA}$ and its optimised derivatives for amber suppression and quadruplet (UAGA) decoding.

Top row: maps of wild type tRNA(Pyl)$_{CUA}$ and its two variants, tRNA(M15)$_{CUA}$ and tRNA(C15)$_{CUA}$ with optimised scaffold. Their quadruplet counterparts are illustrated on the second row. Hybrid tRNAs combining optimised scaffolds with evolved anticodon loops UAGA-1 and M7 are shown on the bottom two rows. Mutations introduced to tRNA(Pyl) scaffold by Serfling *et al.* are coloured orange. Nucleotide differences of UAGA-1 and M7 anticodon loops from that of tRNA(Pyl)$_{UCUA}$ are coloured blue. Anticodons are bold and quadruplet anticodons are shown in red and bold.

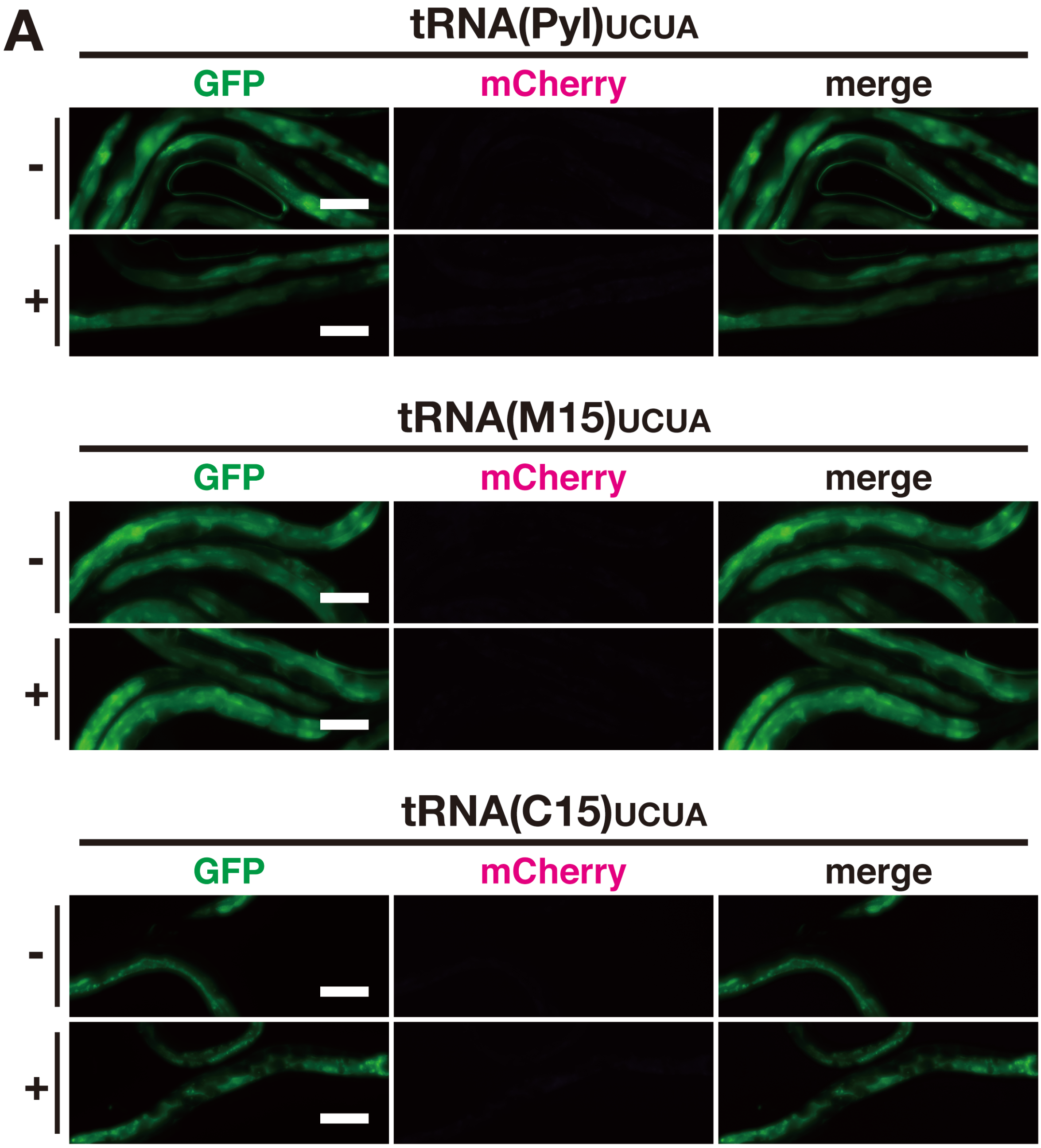

Supplementary Figure 2A: Magnified versions of images shown in Figure 2C of strains expressing tRNA(Pyl)$_{UCUA}$, tRNA(M15)$_{UCUA}$ and tRNA(C15)$_{UCUA}$.
Scale bar = 100 µm.

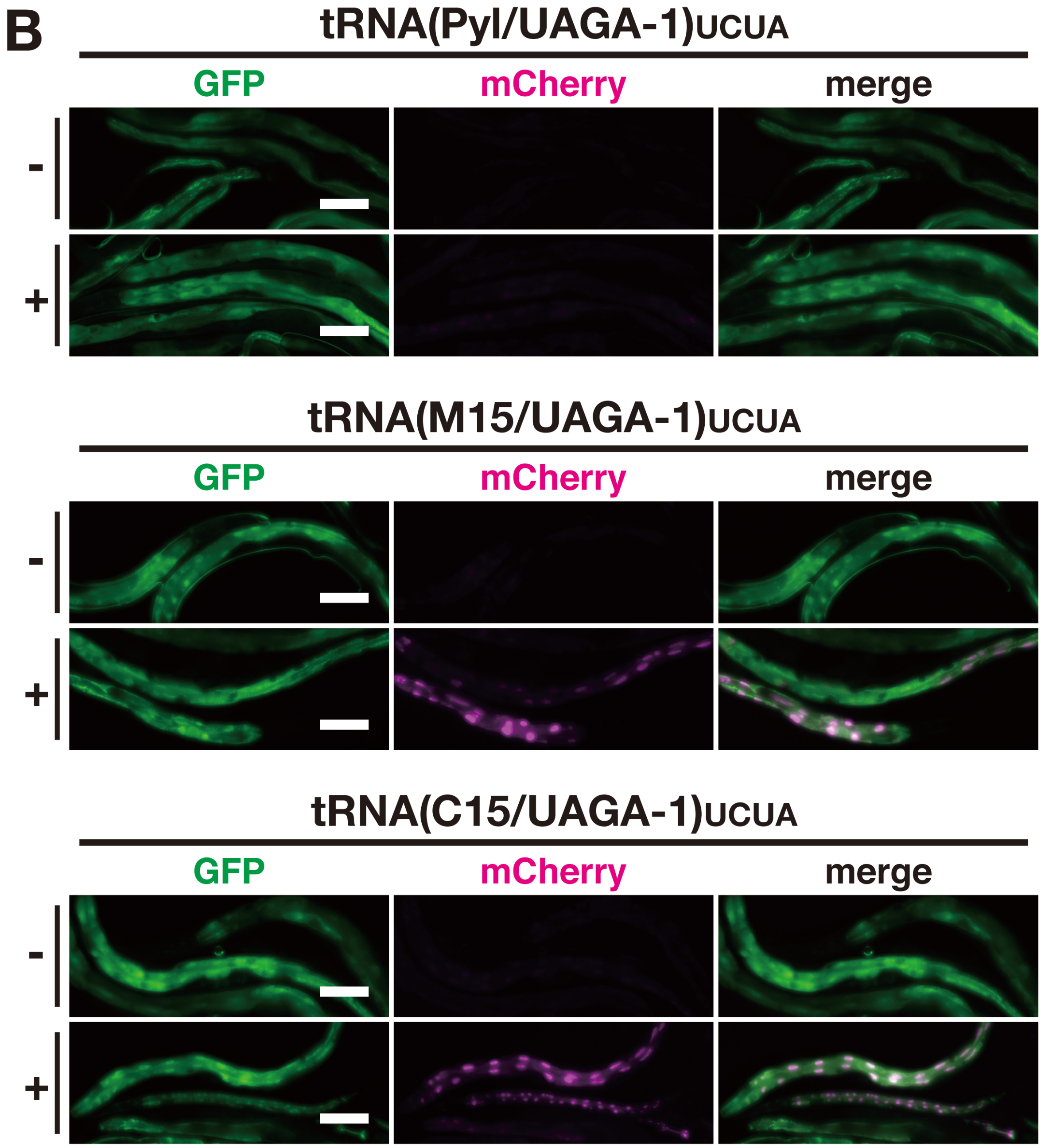

Supplementary Figure 2B: Magnified versions of images shown in Figure 2C of strains expressing tRNA(Pyl/UAGA-1)$_{UCUA}$, tRNA(M15/UAGA-1)$_{UCUA}$ and tRNA(C15/UAGA-1)$_{UCUA}$. Scale bar = 100 µm.

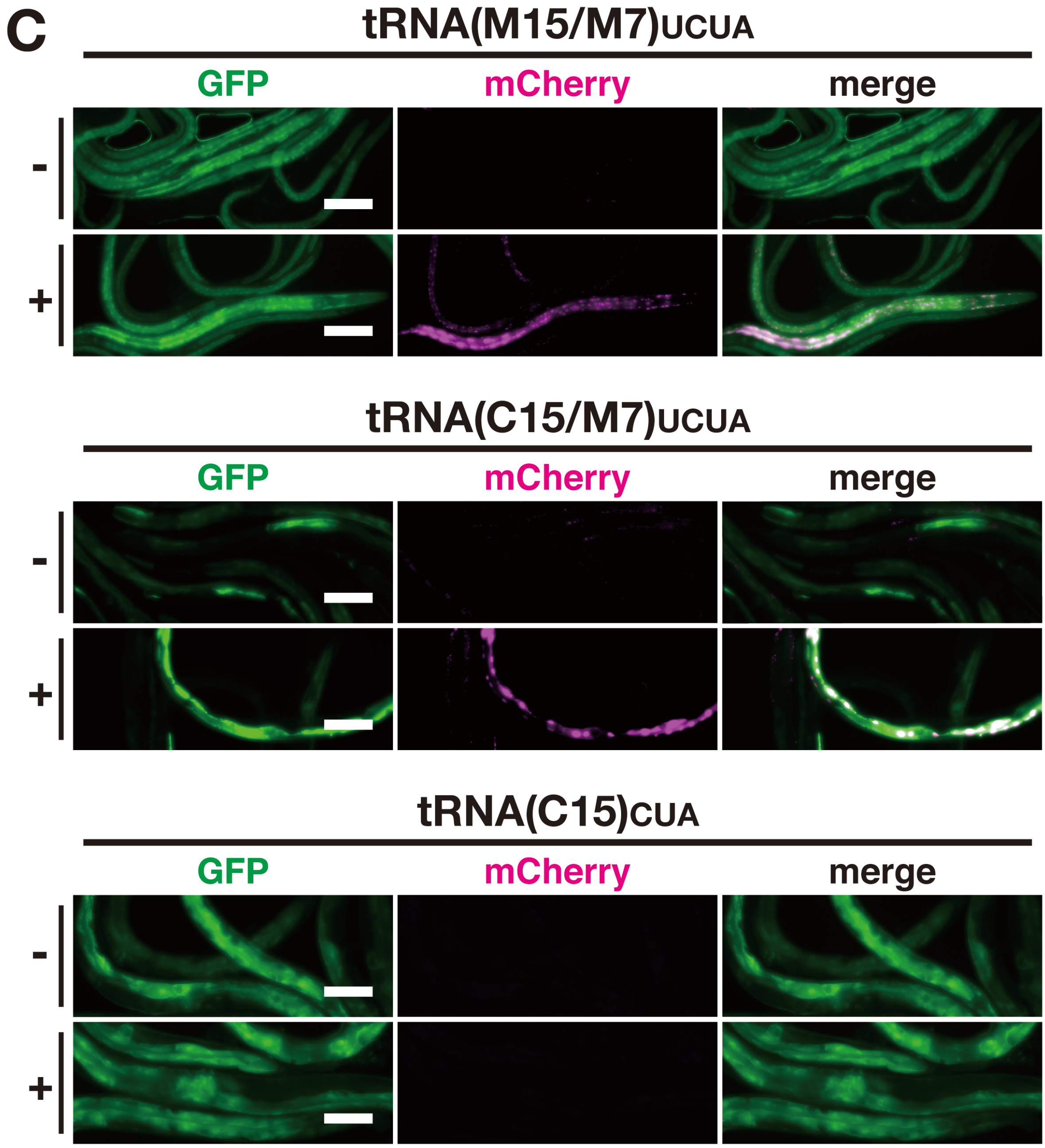

Supplementary Figure 2C: Magnified versions of images shown in Figure 2C of strains expressing tRNA(M15/M7)$_{UCUA}$, tRNA(C15/M7)$_{UCUA}$ and tRNA(C15)$_{CUA}$.
Scale bar = 100 µm.

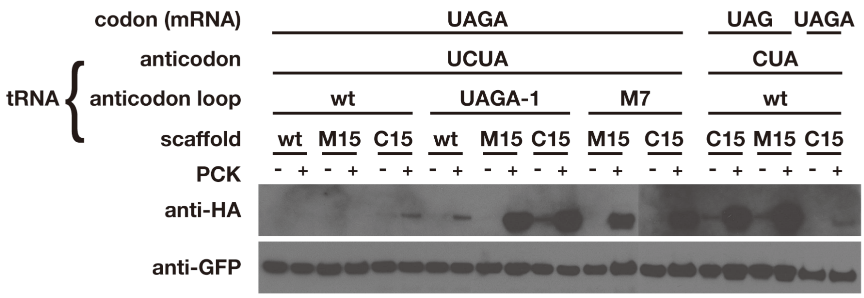

Supplementary Figure 3: Extended exposure of western blot shown in Figure 2D.

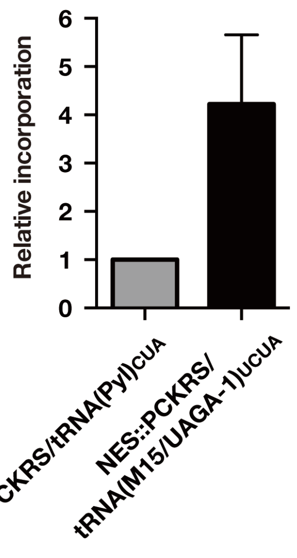

Supplementary Figure 4: Quantitative western blots comparing quadruplet to non-optimised triplet decoding.

Quantitative western blots of the most efficient quadruplet system (consisting of NES::PCKRS and tRNA(M15/UAGA-1)$_{UCUA}$) compared to the non-optimised triplet system (consisting of PCKRS and tRNA(Pyl)$_{CUA}$). Relative incorporation was determined by dividing the intensity of the full-length product bands for each experiment with the mean intensity of the truncated GFP products. Incorporation was performed in the presence of 1mM PCK. The full-length product was detected using anti-HA antibody, the truncated product was detected using anti-GFP antibody. Two independent lines were assayed for each condition. For PCKRS / tRNA(Pyl)$_{CUA}$ each line was independently grown on PCK twice, NES::PCKRS / tRNA(M15/UAGA-1)$_{UCUA}$ was grown independently on PCK three times. Each sample was blotted and measured twice. Error bars show the standard error of the mean.

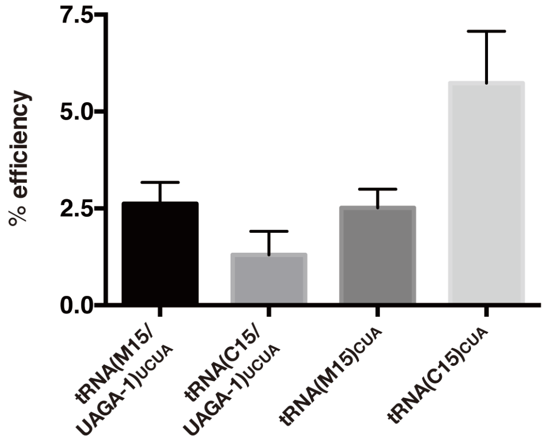

Supplementary Figure 5: PCK incorporation efficiency of the best triplet and quadruplet decoding systems.

Quantitative western blots of the two best quadruplet systems (consisting of NES::PCKRS and either tRNA(M15/UAGA-1)$_{UCUA}$ or tRNA(C15/UAGA-1)$_{UCUA}$) and two best triplet systems (consisting of PCKRS and either tRNA(M15)$_{CUA}$ or tRNA(C15)$_{CUA}$). Efficiency of each group was determined by dividing the intensity of the full-length product bands with the intensity of the truncated GFP product. Incorporation was performed in the presence of 1mM PCK. Both types of products were detected using anti-GFP antibody. Each sample was blotted and measured twice. Error bars show the standard error of the mean.

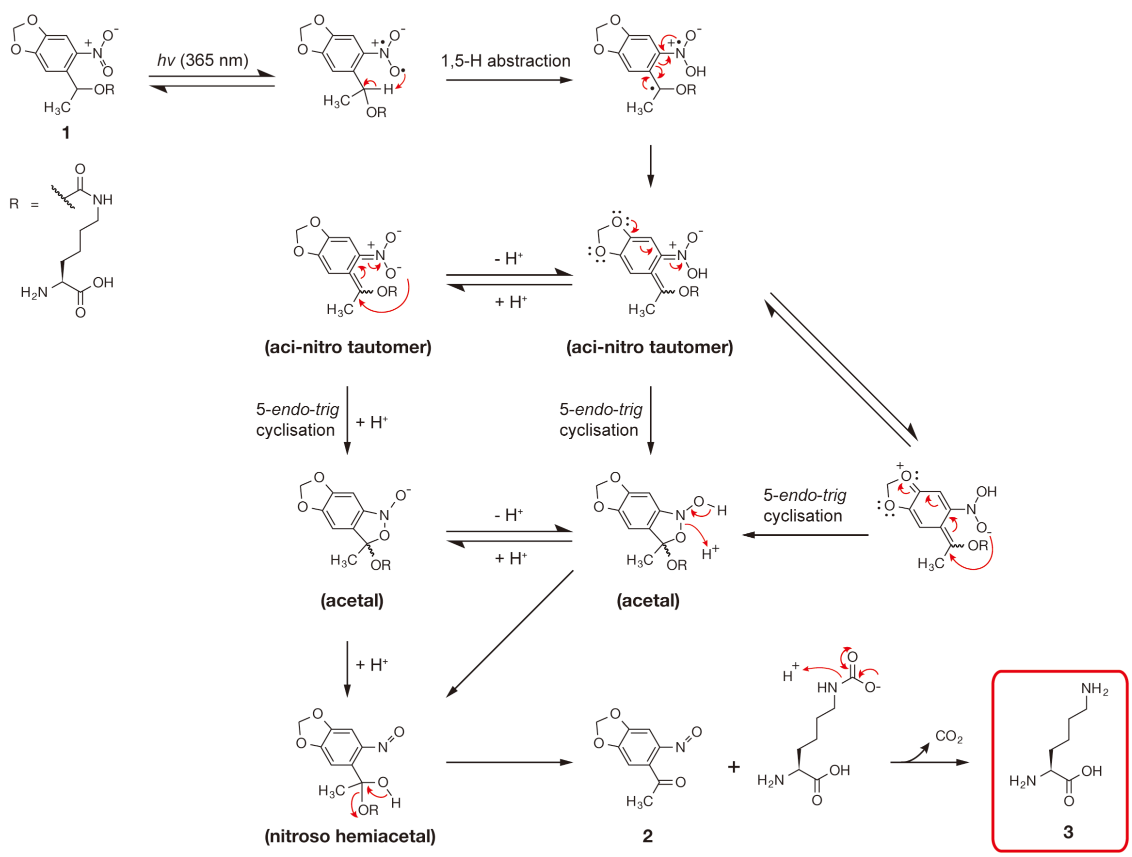

Supplementary Figure 6: PCK uncaging mechanism.
Illumination of photo-caged compound PCK **1** with 365nm light results in the products nitrosoacetophenone **2**, carbon dioxide and lysine **3**.

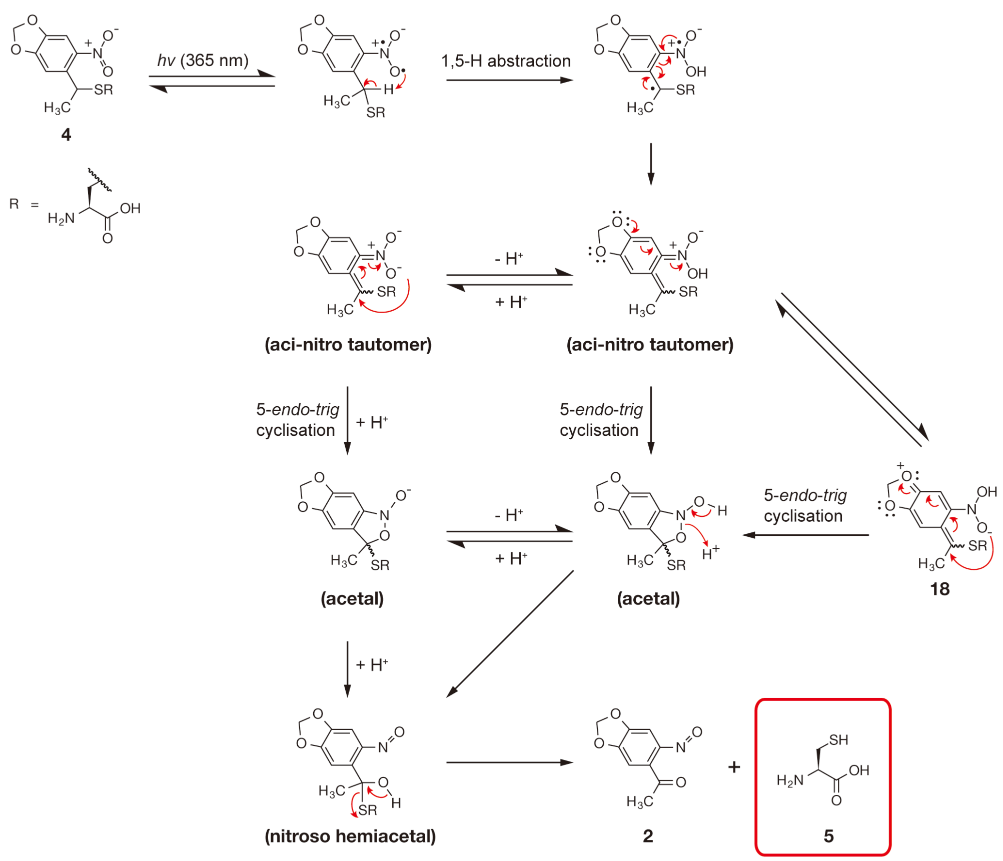

Supplementary Figure 7: PCC uncaging mechanism.
Illumination of 365nm light rapidly photo-cleave PCC **4** to generate nitrosoacetophenone **2** and cysteine **5** at room temperature.

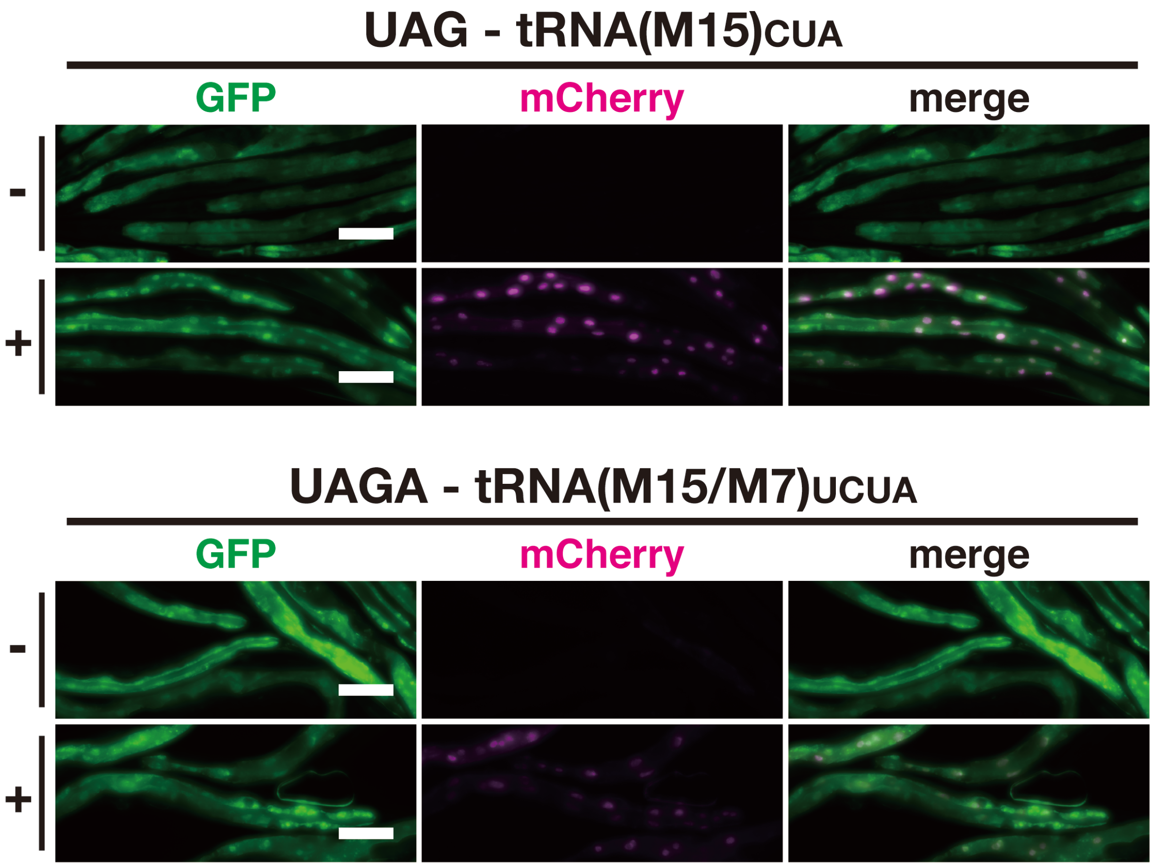

Supplementary Figure 8: Magnified versions of images shown in Figure 4C of strains expressing tRNA(M15)$_{CUA}$ with the UAG fluorescent reporter (top), and tRNA(M15/M7)$_{UCUA}$ UAGA fluorescent reporter (bottom).
Scale bar = 100 µm.

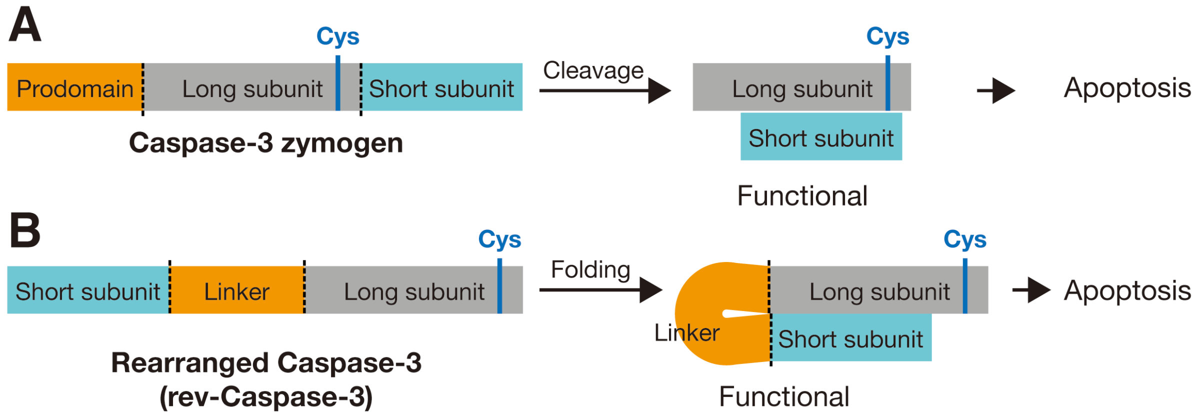

Supplementary Figure 9: Schematic of Caspase-3 activation.
(A) Human Caspase-3 zymogen consists of a prodomain (orange), long subunit (grey) and short subunit (cyan), all separated by caspase cleavage sites (dashed lines). Cleavage removes the prodomain and enables assembly of the long and short subunits to form active caspase. **(B)** Caspases can be expressed in a constitutively active form by reversing the subunit order in the polypeptide.

Supplementary Table 1 - Sequence of tRNA variants

tRNA expression cassette for (the cassette for tRNA(Pyl)$_{UCUA}$ plasmid is shown as an example). The *rpr-1p* promoter sequence is underlined and tRNA(Pyl)$_{UCUA}$ nucleotides are shaded. Below the sequences of all tRNA variants used are shown. Anticodons are marked in red and bold. Nucleotides changes as compared to tRNA(Pyl)$_{UCUA}$ are underlined.

Name	Sequence
tRNA(Pyl)$_{UCUA}$ expression cassette	CGATTTCGGCTAAAAAATAGCGAAAAAACATCAAATTTGTATTAAAAAGAAGCAGTTGAAATTTTGAGTGAGGCTCAGAGACTACAAACTACAAAAAGGCTCAGCCTCAACCAATTTTTAGTGTTAAAAATTTGATTTTTTCAGTTAAAAACGATGGTTTTTGATGCTTTTTACCTATTTTACAGATAGAAAATTTTAAATTTCGCAAAAATCTTTAAAAATAAACTTTTTTGTTTGGTTTTCGCTCGAAAATAGCTTTAAATTTGCAGTTTTTCTCTCAAAAACTCTAAATTTCATGCGTTCCACGTGGCTTTCTATCAATTTAAATACTAATATTAATATTTTCTTCAAAATCTGCCACGTTACAGCCTGCCATAGCCTTCTGAAACTCTACAATTCCCCTCTAATCACTAGCGCCGCCTCCCCGCACACTCAACACAGCGAGCGGCGGAACCCGCGGGGTGTCGGCCGCTCGGACACGCTCCCGTATATAACGACGCGGCGCGCGTCAAGTTGTATGGAAACCTGATCATGTAGATCGAATGGACT**TCTA**AATCCGTTCAGCCGGGTTAGATTCCCGGGGTTTCCGAATTTTTTGTTTTTTAAGTAGTAATATAATACAATTTAATTCAAAATTACACGCAAAAATTTAATAAAGTAGTCCAAAATGCTAATCGTGTGAAAAAATG
tRNA(M15)$_{CUA}$	GGAAACCTGGTCAGGGAGACCGAACGGACT**CTA**AATCCGTTCAGCCGGGTTCGATTCCCGGGGTTTCCG
tRNA(C15)$_{CUA}$	GGGAGAGTGGCCAAGGTGGCCGTGTTGACT**CTA**AATCAACACAGGGGGGTTCGATTCCCCCCTCTCCCG
tRNA(M15)$_{UCUA}$	GGAAACCTGGTCAGGGAGACCGAACGGACT**TCTA**AATCCGTTCAGCCGGGTTCGATTCCCGGGGTTTCCG
tRNA(C15)$_{UCUA}$	GGGAGAGTGGCCAAGGTGGCCGTGTTGACT**TCTA**AATCAACACAGGGGGGTTCGATTCCCCCCTCTCCCG
tRNA(Pyl/UAGA-1)$_{UCUA}$	GGAAACCTGATCATGTAGATCGAATGGGCT**TCTA**ATCCTGTTCAGCCGGGTTAGATTCCCGGGGTTTCCG
tRNA(M15/UAGA-1)$_{UCUA}$	GGAAACCTGGTCAGGGAGACCGAACGGGCT**TCTA**ATCCTGTTCAGCCGGGTTCGATTCCCGGGGTTTCCG
tRNA(C15/UAGA-1)$_{UCUA}$	GGGAGAGTGGCCAAGGTGGCCGTGTGGGCT**TCTA**ATCCTACACAGGGGGGTTCGATTCCCCCCTCTCCCG
tRNA(M15/M7)$_{UCUA}$	GGAAACCTGGTCAGGGAGACCGAAGGGGCT**TCTA**ATCCGCTTCAGCCGGGTTCGATTCCCGGGGTTTCCG
tRNA(C15/M7)$_{UCUA}$	GGGAGAGTGGCCAAGGTGGCCGTGGGGGCT**TCTA**ATCCGCCACAGGGGGGTTCGATTCCCCCCTCTCCCG

Supplementary Table 2 - Transgenic *C. elegans* Strains

Name	Description	Plasmid 1	Plasmid 2	Plasmid 3	Plasmid 4
SGR46	*greEx33[sur-5p::Smad4-NES::PCKRS; rpr-1p::tRNA(C15)$_{CUA}$; rps-0p::GFP(TAG)::mCherry::HA::egl-13 NLS]*	SE170	SE150	SG88	/
SGR48	*greEx35[sur-5p::Smad4-NES::PCKRS; rpr-1p::tRNA(C15)$_{CUA}$; rps-0p::GFP(TAG)::mCherry::HA::egl-13 NLS]*	SE170	SE150	SG88	/
SGR51	*greEx38[glr-1p::Smad4-NES::PCKRS; rpr-1p::tRNA(C15)$_{CUA}$; glr-1p::Cre K201TAG::SL2::GFP; glr-1p::loxP::B-gal terminator + loxP::ChR2::mKate2]*	SE174	SE150	ZS11	IR361
SGR69	*greEx55[sur-5p::Smad4-NES::PCKRS; rpr-1p::tRNA(M15)$_{CUA}$; rps-0p::GFP(TAG)::mCherry::HA::egl-13 NLS]*	SE170	SE149	SG88	/
SGR70	*greEx56[sur-5p::Smad4-NES::PCKRS; rpr-1p::tRNA(M15)$_{CUA}$; rps-0p::GFP(TAG)::mCherry::HA::egl-13 NLS]*	SE170	SE149	SG88	/

SGR71	*greEx57[sur-5p::Smad4-NES::PCKRS; rpr-1p::tRNA(M15)$_{UCUA}$; rps-0p::GFP(TAGA)::mCherry::HA::egl-13 NLS]*	SE170	SE292	KB184	/
SGR72	*greEx58[sur-5p::Smad4-NES::PCKRS; rpr-1p::tRNA(M15)$_{UCUA}$; rps-0p::GFP(TAGA)::mCherry::HA::egl-13 NLS]*	SE170	SE292	KB184	/
SGR73	*greEx59[sur-5p::Smad4-NES::PCKRS; rpr-1p::tRNA(C15)$_{UCUA}$; rps-0p::GFP(TAGA)::mCherry::HA::egl-13 NLS]*	SE170	SE296	KB184	/
SGR74	*greEx60[sur-5p::Smad4-NES::PCKRS; rpr-1p::tRNA(C15)$_{UCUA}$; rps-0p::GFP(TAGA)::mCherry::HA::egl-13 NLS]*	SE170	SE296	KB184	/
SGR75	*greEx61[sur-5p::Smad4-NES::PCKRS; rpr-1p::tRNA(Pyl/UAGA-1)$_{UCUA}$; rps-0p::GFP(TAGA)::mCherry::HA::egl-13 NLS]*	SE170	SE301	KB184	/
SGR76	*greEx62[sur-5p:Smad4-NES::PCKRS; rpr-1p::tRNA(Pyl/UAGA-1)$_{UCUA}$; rps-0p::GFP(TAGA)::mCherry::HA::egl-13 NLS]*	SE170	SE301	KB184	/
SGR77	*greEx63[sur-5p::Smad4-NES::PCKRS; rpr-1p::tRNA(M15/UAGA-1)$_{UCUA}$; rps-0p::GFP(TAGA)::mCherry::HA::egl-13 NLS]*	SE170	SE302	KB184	/
SGR78	*greEx64[sur-5p::Smad4-NES::PCKRS; rpr-1p::tRNA(M15/UAGA-1)$_{UCUA}$; rps-0p::GFP(TAGA)::mCherry::HA::egl-13 NLS]*	SE170	SE302	KB184	/
SGR79	*greEx65[sur-5p::Smad4-NES::PCKRS; rpr-1p::tRNA(C15/UAGA-1)$_{UCUA}$; rps-0p::GFP(TAGA)::mCherry::HA::egl-13 NLS]*	SE170	SE303	KB184	/
SGR80	*greEx66[sur-5p::Smad4-NES::PCKRS; rpr-1p::tRNA(C15/UAGA-1)$_{UCUA}$; rps-0p::GFP(TAGA)::mCherry::HA::egl-13 NLS]*	SE170	SE303	KB184	/
SGR81	*greEx67[sur-5p::Smad4-NES::PCKRS; rpr-1p::tRNA(Pyl)$_{UCUA}$; rps-0p::GFP(TAGA)::mCherry::HA::egl-13 NLS]*	SE170	SE304	KB184	/
SGR82	*greEx68[sur-5p::Smad4-NES::PCKRS; rpr-1p::tRNA(Pyl)$_{UCUA}$; rps-0p::GFP(TAGA)::mCherry::HA::egl-13 NLS]*	SE170	SE304	KB184	/
SGR83	*greEx69[sur-5p::Smad4-NES::PCKRS; rpr-1p::tRNA(C15)$_{CUA}$; rps-0p::GFP(TAGA)::mCherry::HA::egl-13 NLS]*	SE170	SE150	KB184	/
SGR84	*greEx70[sur-5p::Smad4-NES::PCKRS; rpr-1p::tRNA(C15)$_{CUA}$; rps-0p::GFP(TAGA)::mCherry::HA::egl-13 NLS]*	SE170	SE150	KB184	/
SGR85	*greEx71[sur-5p::Smad4-NES::PCKRS; rpr-1p::tRNA(M15/M7)$_{UCUA}$; rps-0p::GFP(TAGA)::mCherry::HA::egl-13 NLS]*	SE170	SE290	KB184	/
SGR86	*greEx72[sur-5p::Smad4-NES::PCKRS; rpr-1p::tRNA(M15/M7)$_{UCUA}$; rps-0p::GFP(TAGA)::mCherry::HA::egl-13 NLS]*	SE170	SE290	KB184	/
SGR87	*greEx73[sur-5p::Smad4-NES::PCKRS; rpr-1p::tRNA(C15/M7)$_{UCUA}$; rps-0p::GFP(TAGA)::mCherry::HA::egl-13 NLS]*	SE170	SE294	KB184	/
SGR88	*greEx74[sur-5p::Smad4-NES::PCKRS; rpr-1p::tRNA(C15/M7)$_{UCUA}$; rps-0p::GFP(TAGA)::mCherry::HA::egl-13 NLS]*	SE170	SE294	KB184	/
SGR89	*greEx75[glr-1p::Smad4-NES::PCKRS; rpr-1p::tRNA(M15/M7)$_{UCUA}$; glr-1p::Cre*	SE174	SE290	SE368	IR361

	K201TAGA::SL2::GFP; glr-1p::loxP::B-gal terminator + loxP::ChR2::mKate2]				
SGR90	*greEx76[glr-1p::Smad4-NES::PCKRS; rpr-1p::tRNA(M15/UAGA-1)*$_{UCUA}$*; glr-1p::Cre K201TAGA::SL2::GFP; glr-1p::loxP::B-gal terminator + loxP::ChR2::mKate2]*	SE174	SE302	SE368	IR361
SGR91	*greEx77[sur-5p::Smad4-NES::PCCRS; rpr-1p::tRNA(M15)*$_{CUA}$*; rps-0p::GFP(TAG)::mCherry::HA::egl-13 NLS]*	SE367	SE149	SG88	/
SGR92	*greEx78[sur-5p::Smad4-NES::PCCRS; rpr-1p::tRNA(M15)*$_{CUA}$*; rps-0p::GFP(TAG)::mCherry::HA::egl-13 NLS]*	SE367	SE149	SG88	/
SGR93	*greEx79[sur-5p::Smad4-NES::PCCRS; rpr-1p::tRNA(M15/M7)*$_{UCUA}$*; rps-0p::GFP(TAGA)::mCherry::HA::egl-13 NLS]*	SE367	SE290	KB184	/
SGR94	*greEx80[sur-5p::Smad4-NES::PCCRS; rpr-1p::tRNA(M15/M7)*$_{UCUA}$*; rps-0p::GFP(TAGA)::mCherry::HA::egl-13 NLS]*	SE367	SE290	KB184	/
SGR95	*greEx81[mec-4p::Smad4-NES::PCCRS::SL2::CFP; rpr-1p::tRNA(M15/M7)*$_{UCUA}$*; mec-4p::rev-Caspase-3(CeOpt) C271TAGA]*	ZX334	SE290	ZX360	/

Supplementary Table 3.1 - Expression plasmids

Name	Description	Notes
IR361	*glr-1p::loxP::B-gal 3'UTR::loxP::ChR2-mKate2::let-858 3'UTR*	(1)
KB184	*rps-0p::GFP(TAGA)::mCherry::HA::egl-13 NLS::unc-54 3'UTR*	SG576 + EH5 + SE187; pDEST R4-R3 II
SE170	*sur-5p::Smad4-NES::Mm PCKRS CeOpt::let-858 3'UTR*	(1)
SE174	*glr-1p::Smad4-NES::Mm PCKRS CeOpt::let-858 3' UTR*	(1)
SE367	*sur-5p::Smad4-NES::Mm PCCRS CeOpt::let-858 3'UTR*	SE72 + KB124 + SG606; IR98
SE368	*glr-1p::PC-Cre K201TAGA::SL2::GFP::let-858 3'UTR*	IR157 + LD441 + IR182; pDEST R4-R3 II
SG88	*rps-0p::GFP(TAG)::mCherry::HA::egl-13 NLS::unc-54 3'UTR*	(2)
ZX334	*mec-4p::Smad4-NES::Mm PCCRS CeOpt::SL2::GFP::let858 3'UTR*	LD400 + KB124 + ZX114; pDEST R4-R3 II
ZX360	*mec-4p::rev-Casp-3(CeOpt) C135TAGA::unc54 3'UTR*	LD400 + ZX339 + ZX358; SE82

Supplementary Table 3.2 - Destination Vectors

Name	Description	Notes
IR98	*pDEST rps-0p::HygR::unc-54 3'UTR*	(3)
SE82	*pDEST wars-*	*rps-0*p of IR98 was replaced by *wars-1*p amplified from genomic DNA

	1p::HygR::unc-54 3'UTR	with primers *wars-1*p attB1R (GGGGACTGCTTTTTTGTACAAACTTGGCTGTGTTGAACCCTGAAAAAAATAAATTGGGG) & *wars-1*p attB4F (GGGGACAACTTTGTATAGAAAAGTTGactagtAAGAGCCACCACCGAAATAGATG)

Supplementary Table 3.3 - pENTR P4-P1r Vectors

Name	Description	Notes
IR157	*glr-1p*	(1)
LD400	*mec-4p*	Cloned from genomic DNA with primers attB4 *mec-4*p F (GGGGACAACTTTGTATAGAAAAGTTGAACTGCCAATCTGTGCAAATTCAGG) & attB1r *mec-4*p R (GGGGACTGCTTTTTTGTACAAACTTGTCTATAACTTGATAGCGATAAAAAAAATAGCATTAGCAAACTG)
SE72	*sur-5p*	(1)
SG576	*rps-0p*	(4)

Supplementary Table 3.4 - pENTR 221 Vectors

Name	Description	Notes
EH5	*GFP-TAGA*	Made from SG4 (2) using primers 457 (ACGAGCTCTACAAGGGAGGATTTaAACCCAGCTTTCTTGTACAA) & 462 (ACGAGCTCTACAAGGGAGGACTCaAACCCAGCTTTCTTGTACAA)
KB124	*Smad4-NES::PCCRS Mm CeOpt*	The PCC2RS gene described by Nguyen *et al*. (5) synthesised after *C. elegans* optimisation (see Supplementary Table 4), with a Smad4-NES (GCCTGCCCAGTCCCACTCCAACTCCCACCACTCGAGCGTCTCACCCTCGAC) then attached to its N-terminus
LD441	*PC-Cre(K201TAGA)*	Made from SG617 (1) by inserting an A to the 5' of amber codon TAG
SE154	*Smad4-NES::Mm PCKRS CeOpt*	(1)
SG322	*rpr-1p::PylT:: sup-7 3'*	(1)
ZX339	*rev-Caspase-3(C271TAGA)*	Synthesised after *C. elegans* optimisation (see Supplementary Table 4), inserted into pDONR221

Supplementary Table 3.5 - pENTR P2r-P3 Vectors

Name	Description	Notes
IR182	*SL2::GFP::let-858 3'UTR*	(1)
SE149	*rpr-1p::tRNA(M15)$_{CUA}$:: sup-7 3'*	The expression cassette from SG322 was cloned into a P2r-P3 vector and PylT was replaced with tRNA(M15)$_{CUA}$ (sequence: GGAAACCTGGTCAGGGAGACCGAACGGACTCTAAATCCGTTCAGCCGGGTTCGATTCCCGGGGTTTCCG)
SE150	*rpr-1p::tRNA(C15)$_{CUA}$::sup-7 3'*	The expression cassette from SG322 was cloned into a P2r-P3 vector and PylT was replaced with tRNA(C15)$_{CUA}$ (sequence: GGGAGAGTGGCCAAGGTGGCCGTGTTGACTCTAAATCAACACAGGGGGGTTCGATTCCCCCCTCTCCCG) (1)
SE187	*mCherry CeOpt::HA::egl-13 NLS::unc-54 3'UTR*	Sequence of mCherry was optimised for *C. elegans*, HA and NLS from SG88 was fused to the mCherry 3' end by overlap extension PCR
SE290	*rpr-1p::tRNA(M15/M7)*	Made from SE149 using PCR followed by NEBuilder, using primers 602 (CGAAGGGGCTtCTAATCCGCTTCAGCCGGGTTcGATTCC) & 603

	$_{UCUA}$::sup-7 3'	(GCGGATTAGaAGCCCCTTCGgTCTcCcTGAcCAGG)
SE292	*rpr-1p::tRNA(M15)$_{UCUA}$::sup-7 3'*	Made from SE149 using PCR followed by NEBuilder, using primers 606 (AAcGGACTtctaAATCCGTTCAGCCGGGTTcG) & 607 (AACGGATTtagaAGTCCgTTCGgTCTcCcTG)
SE294	*rpr-1p::tRNA(C15/M7)$_{UCUA}$::sup-7 3'*	Made from SE150 using PCR followed by NEBuilder, using primers 610 (TGGGGGCTTCTAATCCGCCACAGGGGGGTTCGATTCC) & 611 (TGGCGGATTAGAAGCCCCCACGGCCACCTTGGCCACTC)
SE296	*rpr-1p::tRNA(C15)$_{UCUA}$::sup-7 3'*	Made from SE150 using PCR followed by NEBuilder, using primers 614 (TGTTGACTtctaAATCAACACAGGGGGGTTC) & 615 (TGTTGATTtagaAGTCAACACGGCCACCTTG)
SE301	*rpr-1p::tRNA(Pyl/UAGA-1/)$_{UCUA}$::sup-7 3'*	The expression cassette from SG322 was cloned into a P2r-P3 vector and the anticodon changed using primers 627 (AATGGGCTtctaATCCTGTTCAGCCGGGTTAGATTCCCGGGGTTTC) & 628 (GAACAGGATtagaAGCCCATTCGATCTACATGATC)
SE302	*rpr-1p::tRNA(M15/UAGA-1)$_{UCUA}$::sup-7 3'*	Made from SE149 using PCR followed by NEBuilder, using primers 629 (AAcGGGCTtctaATCCTGTTCAGCCGGGTTcGATTCCCGGGGTTTC) & 630 (AACAGGATtagaAGCCCgTTCGgTCTcCcTGAcCAGGTTTC)
SE303	*rpr-1p::tRNA(C15/UAGA-1)$_{UCUA}$::sup-7 3'*	Made from SE150 using PCR followed by NEBuilder, using primers 631 (CGTGTGGGCTtctaATCCTACACAGGGGGGTTCGATTC) & 632 (TGTAGGATtagaAGCCCACACGGCCACCTTGGCCACTC)
SE304	*rpr-1p::tRNA(Pyl)$_{UCUA}$::sup-7 3'*	The expression cassette from SG322 was cloned into a P2r-P3 vector with anticodon loop changed, using primers 625 (AATGGACTtCTAAATCCGTTCAGCCGGGTTAG) & 626 (GAACGGATTTAGaAGTCCATTCGATCTACATGATC)
SG304	*GFP::let-858 3'UTR*	(1)
SG606	*let-858 3'UTR*	(1)
ZX114	*SL2::CFP::let858 3'UTR*	The *gpd-2/3* intergenic region SL2 was amplified from genomic DNA and fused to optimised CFP. A *let-858* 3' UTR from SG606 was attached to the 3' end of CFP.
ZX358	*unc-54 3'UTR*	Inserted into pDONR P2R-P3

Supplementary Table 4 - Gene sequences

The codons used to designate the ncAA incorporation site are marked in red. Different DNA elements are shaded in different colours as indicated. Artificial introns are in lowercase.

**Name & Description**	**Sequence**
Mm PCCRS CeOpt. (*Methanosarcina mazei* pyrrolysine aminoacyl tRNA synthetase mutated to recognise photocaged cysteine and codon optimised for expression in *C. elegans*)	ATGGACAAGAAGCCACTCAACACCCTCATCTCCGCCACCGGACTC<br>TGGATGTCCCGTACCGGAACCATCCACAAGATCAAGCACCACGAG<br>GTCTCCCGTTCCAAGATCTACATCGAGATGGCCTGCGGAGACCAC<br>CTCGTCGTCAACAACTCCCGTTCCTCCCGTACCGCCCGTGCCCTC<br>CGTCACCACAAGTACCGTAAGACCTGCAAGCGTTGCCGTGTCTCC<br>GACGAGGACCTCAACAAGTTCCTCACCAAGGCCAACGAGGACCAA<br>ACCTCCGTCAAGGTCAAGGTCGTCTCCGCCCCAACCCGTACCAAG<br>AAGgtaagtttaaacatatatatactaactaaccctgattattta<br>aattttcagGCCATGCCAAAGTCCGTCGCCCGTGCCCCAAAGCCA<br>CTCGAGAACACCGAGGCCGCCCAAGCCCAACCATCCGGATCCAAG<br>TTCTCCCCAGCCATCCCAGTCTCCACCCAAGAGTCCGTCTCCGTC<br>CCAGCCTCCGTCTCCACCTCCATCTCCTCCATCTCCACCGGAGCC<br>ACCGCCTCCGCCCTCGTCAAGGGAAACACCAACCCAATCACCTCC<br>ATGTCCGCCCCAGTCCAAGCCTCCGCCCCAGCCCTCACCAAGTCC<br>CAAACCGACCGTCTCGAGGTCCTCCTCAACCCAAAGGACGAGATC<br>TCCCTCAACTCCGGAAAGCCATTCCGTGAGCTCGAGTCCGAGCTC

	CTCTCCCGTCGTAAGgtaagtttaaacagttcggtactaactaac<br>catacatatttaaattttcagAAGGACCTCCAACAAATCTACGCC<br>GAGGAGCGTGAGAACTACCTCGGAAAGCTCGAGCGTGAGATCACC<br>CGTTTCTTCGTCGACCGTGGATTCCTCGAGATCAAGTCCCCAATC<br>CTCATCCCACTCGAGTACATCGAGCGTATGGGAATCGACAACGAC<br>ACCGAGCTCTCCAAGCAAATCTTCCGTGTCGACAAGAACTTCTGC<br>CTCCGTCCAATGCTCGCCCCAAACCTCTACAACTACCTCCGTAAG<br>CTCGACCGTGCCCTCCCAGACCCAATCAAGATCTTCGAGATCGGA<br>CCATGCTACCGTAAGGAGTCCGACGGAAAGgtaagtttaaacatg<br>attttactaactaactaatctgatttaaattttcagGAGCACCTC<br>GAGGAGTTCACCATGCTCCAGTTCGCCCAAATGGGATCCGGATGC<br>ACCCGTGAGAACCTCGAGTCCATCATCACCGACTTCCTCAACCAC<br>CTCGGAATCGACTTCAAGATCGTCGGAGACTCCTGCATGGTCTAC<br>GGAGACACCCTCGACGTCATGCACGGAGACCTCGAGCTCTCCTCC<br>GCCATGGTCGGACCAATCCCACTCGACCGTGAGTGGGAATCGAC<br>AAGCCATGGATCGGAGCCGGATTCGGACTCGAGCGTCTCCTCAAG<br>GTCAAGCACGACTTCAAGAACATCAAGCGTGCCGCCCGTTCCGAG<br>TCCTACTACAACGGAATCTCCACCAACCTCTAA
Mm PCKRS CeOpt. (*Methanosarcina mazei* pyrrolysine aminoacyl tRNA synthetase mutated to recognise photocaged lysine and codon optimised for expression in *C. elegans*)	ATGGACAAGAAGCCACTCAACACCCTCATCTCCGCCACCGGACTC<br>TGGATGTCCCGTACCGGAACCATCCACAAGATCAAGCACCACGAG<br>GTCTCCCGTTCCAAGATCTACATCGAGATGGCCTGCGGAGACCAC<br>CTCGTCGTCAACAACTCCCGTTCCTCCCGTACCGCCCGTGCCCTC<br>CGTCACCACAAGTACCGTAAGACCTGCAAGCGTTGCCGTGTCTCC<br>GACGAGGACCTCAACAAGTTCCTCACCAAGGCCAACGAGGACCAA<br>ACCTCCGTCAAGGTCAAGGTCGTCTCCGCCCCAACCCGTACCAAG<br>AAGgtaagtttaaacatatatatactaactaaccctgattattta<br>aattttcagGCCATGCCAAAGTCCGTCGCCCGTGCCCCAAAGCCA<br>CTCGAGAACACCGAGGCCGCCCAAGCCCAACCATCCGGATCCAAG<br>TTCTCCCCAGCCATCCCAGTCTCCACCCAAGAGTCCGTCTCCGTC<br>CCAGCCTCCGTCTCCACCTCCATCTCCTCCATCTCCACCGGAGCC<br>ACCGCCTCCGCCCTCGTCAAGGGAAACACCAACCCAATCACCTCC<br>ATGTCCGCCCCAGTCCAAGCCTCCGCCCCAGCCCTCACCAAGTCC<br>CAAACCGACCGTCTCGAGGTCCTCCTCAACCCAAAGGACGAGATC<br>TCCCTCAACTCCGGAAAGCCATTCCGTGAGCTCGAGTCCGAGCTC<br>CTCTCCCGTCGTAAGgtaagtttaaacagttcggtactaactaac<br>catacatatttaaattttcagAAGGACCTCCAACAAATCTACGCC<br>GAGGAGCGTGAGAACTACCTCGGAAAGCTCGAGCGTGAGATCACC<br>CGTTTCTTCGTCGACCGTGGATTCCTCGAGATCAAGTCCCCAATC<br>CTCATCCCACTCGAGTACATCGAGCGTTTCGGAATCGACAACGAC<br>ACCGAGCTCTCCAAGCAAATCTTCCGTGTCGACAAGAACTTCTGC<br>CTCCGTCCAATGCTCTCCCCAAACCTCTGCAACTACATGCGTAAG<br>CTCGACCGTGCCCTCCCAGACCCAATCAAGATCTTCGAGATCGGA<br>CCATGCTACCGTAAGGAGTCCGACGGAAAGgtaagtttaaacatg<br>attttactaactaactaatctgatttaaattttcagGAGCACCTC<br>GAGGAGTTCACCATGCTCAACTTCTGCCAAATGGGATCCGGATGC<br>ACCCGTGAGAACCTCGAGTCCATCATCACCGACTTCCTCAACCAC<br>CTCGGAATCGACTTCAAGATCGTCGGAGACTCCTGCATGGTCTAC<br>GGAGACACCCTCGACGTCATGCACGGAGACCTCGAGCTCTCCTCC<br>GCCGTCGTCGGACCAATCCCACTCGACCGTGAGTGGGGAATCGAC<br>AAGCCATGGATCGGAGCCGGATTCGGACTCGAGCGTCTCCTCAAG<br>GTCAAGCACGACTTCAAGAACATCAAGCGTGCCGCCCGTTCCGAG<br>TCCTACTACAACGGAATCTCCACCAACCTCTAA
GFP(TAG)::mCherry::HA::egl-13 NLS. (GFP, **amber stop codon**, mCherry, HA, *egl-13* NLS)	ATGTCCAAGGGAGAGGAGCTCTTCACCGGAGTCGTCCCAATCCTC<br>GTCGAGCTCGACGGAGACGTCAACGGACACAAGTTCTCCGTCTCC<br>GGAGAGGGAGAGGGAGACGCCACCTACGGAAAGCTCACCCTCAAG<br>TTCATCTGCACCACCGGAAAGCTCCCAGTCCCATGGCCAACCCTC<br>GTCACCACCTTCACCTACGGAGTCCAATGCTTCTCCCGTTACCCA<br>Ggtaagtttaaacatatatatactaactaaccctgattatttaaa<br>ttttcagACCACATGAAGCGTCACGACTTCTTCAAGTCCGCCATG<br>CCAGAGGGATACGTCCAAGAGCGTACCATCTTCTTCAAGGACGAC<br>GGAAACTACAAGACCCGTGCCGAGGTCAAGTTCGAGGGAGACACC<br>CTCGTCAACCGTATCGAGCTCAAGGGAATCGACTTCAAGgtaagt<br>ttaaacagttcggtactaactaaccatacatatttaaattttcag

	GAGGACGGAAACATCCTCGGACACAAGCTCGAGTACAACTACAAC<br>TCCCACAACGTCTACATCATGGCCGACAAGCAAAAGAACGGAATC<br>AAGGTCAACTTCAAGATCCGTCACAACATCGAGGACGGATCTGTC<br>CAACTCGCCGACCACTACCAACAAAACACCCCAATCGGAGACGGA<br>CCAGTCCTCCTCCCAGgtaagtttaaacatgattttactaactaa<br>ctaatctgatttaaattttcagACAACCACTACCTCTCCACCCAA<br>TCCGCCCTCTCCAAGGACCCAAACGAGAAGCGTGACCACATGGTC<br>CTCAAGGAGTTCGTCACCGCCGCCGGAATCACCCACGGAATGGAC<br>GAGCTCTACAAGGGAGGA**TAG**GGCGCGCCAGGCCGGCCAAACCCA<br>GCTTTCTTGTACAAAGTGGCCATGGTCTCAAAGGGTGAAGAAGAT<br>AACATGGCAATTATTAAAGAGTTTATGCGTTTCAAGGTGCATATG<br>GAGGGATCTGTCAATGGGCATGAGTTTGAAATTGAAGGTGAAGGA<br>GAAGGCCGACCATATGAGGGAACACAAACCGCAAAACTAAAGgta<br>agtttaaacatatatatactaactaaccctgattatttaaatttt<br>cagGTAACTAAAGGCGGACCATTACCATTCGCCTGGGACATCCTC<br>TCTCCACAGTTCATGTATGGAAGTAAAGCTTATGTTAAACATCCG<br>GCAGATATACCAGATTATTTGAAACTTTCATTCCCGGAGGGTTTT<br>AAGTGGGAACGCGTAATGAATTTTGAAGACGGAGGAGTTGTTACA<br>GTGACGCAAGACTCAAGgtaagtttaaacagttcggtactaacta<br>accatacatatttaaattttcagCCTCCAAGATGGAGAATTTATT<br>TATAAAGTCAAACTTCGAGGAACGAATTTCCCCTCGGATGGACCT<br>GTTATGCAGAAGAAGACTATGGGATGGGAAGCTTCAAGTGAAAGA<br>ATGTACCCTGAAGACGGTGCTCTTAAGGGAGAGATTAAACAACGT<br>CTTAAATTGAAAGATGGAGGACATTACGATGCTGAGgtaagttta<br>aacatgattttactaactaactaatctgatttaaattttcagGTG<br>AAGACAACTTACAAAGCCAAAAAACCAGTTCAGCTGCCAGGAGCG<br>TACAATGTTAATATTAAACTGGATATCACCTCCCACAACGAGGAT<br>TACACTATCGTTGAGCAATATGAAAGAGCTGAAGGGCGGCACTCG<br>ACAGGTGGCATGGATGAATTGTATAAGTACCCATATGATGTCCCA<br>GACTACGCTATGAGCCGTAGACGAAAAGCGAATCCGACAAAACTG<br>AGTGAAAACGCGAAGAAGCTTGCCAAGGAAGTTGAAAATTAA
GFP(TAGA)::mCherry::HA::egl-13 NLS. (GFP, **quadruplet codon TAGA**, mCherry, HA, *egl-13* NLS	ATGTCCAAGGGAGAGGAGCTCTTCACCGGAGTCGTCCCAATCCTC<br>GTCGAGCTCGACGGAGACGTCAACGGACACAAGTTCTCCGTCTCC<br>GGAGAGGGAGAGGGAGACGCCACCTACGGAAAGCTCACCCTCAAG<br>TTCATCTGCACCACCGGAAAGCTCCCAGTCCCATGGCCAACCCTC<br>GTCACCACCTTCACCTACGGAGTCCAATGCTTCTCCCGTTACCCA<br>Ggtaagtttaaacatatatatactaactaaccctgattatttaaa<br>ttttcagACCACATGAAGCGTCACGACTTCTTCAAGTCCGCCATG<br>CCAGAGGGATACGTCCAAGAGCGTACCATCTTCTTCAAGGACGAC<br>GGAAACTACAAGACCCGTGCCGAGGTCAAGTTCGAGGGAGACACC<br>CTCGTCAACCGTATCGAGCTCAAGGGAATCGACTTCAAGGTAAGT<br>TTAAACAGTTCGGTACTAACTAACCATACATATTTAAATTTTCAG<br>GAGGACGGAAACATCCTCGGACACAAGCTCGAGTACAACTACAAC<br>TCCCACAACGTCTACATCATGGCCGACAAGCAAAAGAACGGAATC<br>AAGGTCAACTTCAAGATCCGTCACAACATCGAGGACGGATCTGTC<br>CAACTCGCCGACCACTACCAACAAAACACCCCAATCGGAGACGGA<br>CCAGTCCTCCTCCCAGgtaagtttaaacatgattttactaactaa<br>ctaatctgatttaaattttcagACAACCACTACCTCTCCACCCAA<br>TCCGCCCTCTCCAAGGACCCAAACGAGAAGCGTGACCACATGGTC<br>CTCAAGGAGTTCGTCACCGCCGCCGGAATCACCCACGGAATGGAC<br>GAGCTCTACAAGGGAGGA**TAGA**AACCCAGCTTTCTTGTACAAAGT<br>GGGAATGGTCTCCAAGGGAGAGGAGGACAACATGGCCATCATCAA<br>GGAGTTCATGCGTTTCAAGGTCCACATGGAGGGATCCGTCAACGG<br>ACACGAGTTCGAGATCGAGGGAGAGGGAGAGGGACGTCCATACGA<br>GGGAACCCAAACCGCCAAGCTCAAGGTCACCAAGGTAAGTTTAAT<br>CATATATATACTAACTAACCCTGATTATTTAAATTTTCAGGGAGG<br>ACCACTCCCATTCGCCTGGGACATCCTCTCCCCACAATTCATGTA<br>CGGATCCAAGGCCTACGTCAAGCACCCAGCCGACATCCCAGACTA<br>CCTCAAGCTCTCCTTCCCAGAGGGATTCAAGTGGGAGCGTGTCAT<br>GAACTTCGAGGACGGAGGAGTCGTCACCGTCACCCAAGACTCCTC<br>CCTCCAAGACGGAGAGTTCATCTACAAGGTAAGTTTAAACAGTTC<br>GGTACTAACTAACCATACATATTTAAATTTTCAGGTCAAGCTCCG<br>TGGAACCAACTTCCCATCCGACGGACCAGTCATGCAAAAGAAGAC

	CATGGGATGGGAGGCCTCCTCCGAGCGTATGTACCCAGAGGACGG<br>AGCCCTCAAGGGAGAGATCAAGCAACGTCTCAAGCTCAAGgtaag<br>tttaaacatgattttactaactaactaatctgatttaattttca<br>gGACGGAGGACACTACGACGCCGAGGTCAAGACCACCTACAAGGC<br>CAAGAAGCCAGTCCAACTCCCAGGAGCCTACAACGTCAACATCAA<br>GCTCGACATCACCTCCCACAACGAGGACTACACCATCGTCGAGCA<br>ATACGAGCGTGCCGAGGGACGTCACTCCACCGGAGGAATGGACGA<br>GCTCTACAAGTACCCATACGACGTCCCAGACTACGCCATGTCCCG<br>TCGTCGAAAGGCCAACCCAACCAAGCTCTCCGAGAACGCCAAGAA<br>GCTCGCCAAGGAGGTCGAGAAC
PC-Cre(K201TAG). (*C. elegans* codon optimised Cre recombinase with a **TAG codon** in place of K201)	ATGGGGGCGCCAAAAAAGAAGAGGAAAGTATCGAATTTGCTCACC<br>GTCCACCAAAACCTCCCAGCCCTCCCAGTCGACGCCACCTCCGAC<br>GAGGTCCGTAAGAACCTCATGGACATGTTCCGTGACCGTCAAGCC<br>TTCTCCGAGCACACCTGGAAGATGCTCCTCTCCGTCTGCCGTTCC<br>TGGGCCGCCTGGTGCAAGCTCAACAACCGTAAGTGGTTCCCAGCC<br>GAGCCAGAGGACGTCCGTGACTACCTCCTCTACCTCCAAGCCCGT<br>GGACTCGCCGTCAAGgtaagtttaaacatatatatactaactaac<br>cctgattatttaaattttcagACCATCCAACAACACCTCGGACAA<br>CTCAACATGCTCCACCGTCGTTCCGGACTCCCACGTCCATCCGAC<br>TCCAACGCCGTCTCCCTCGTCATGCGTCGTATCCGTAAGGAGAAC<br>GTCGACGCCGGAGAGCGTGCCAAGCAAGCCCTCGCCTTCGAGCGT<br>ACCGACTTCGACCAAGTCCGTTCCCTCATGGAGAACTCCGACCGT<br>TGCCAAGACATCCGTAACCTCGCCTTCCTCGGAATCGCCTACAAC<br>ACCCTCCTCCGTATCGCCGAGATCGCCCGTATCCGTGTCAAGGTA<br>AGTTTAAACAGTTCGGTACTAACTAACCATACATATTTAAATTTT<br>CAGGACATCTCCCGTACCGACGGAGGACGTATGCTCATCCACATC<br>GGACGTACC**TAG**ACCCTCGTCTCCACCGCCGGAGTCGAGAAGGCC<br>CTCTCCCTCGGAGTCACCAAGCTCGTCGAGCGTTGGATCTCCGTC<br>TCCGGAGTCGCCGACGACCCAAACAACTACCTCTTCTGCCGTGTC<br>CGTAAGgtaagtttaaacatgattttactaactaactaatctgat<br>ttaaattttcagAACGGAGTCGCCGCCCCATCCGCCACCTCCCAA<br>CTCTCCACCCGTGCCCTCGAGGGAATCTTCGAGGCCACCCACCGT<br>CTCATCTACGGAGCCAAGGACGACTCCGGACAACGTTACCTCGCC<br>TGGTCCGGACACTCCGCCCGTGTCGGAGCCGCCCGTGACATGGCC<br>CGTGCCGGAGTCTCCATCCCAGAGATCATGCAAGCCGGAGGATGG<br>ACCAACGTCAACATCGTCATGAACTACATCCGTAACCTCGACTCC<br>GAGACCGGAGCCATGGTCCGTCTCCTCGAGGACGGAGACTAA
PC-Cre(K201TAGA). (*C. elegans* codon optimised Cre recombinase with a **TAGA quadruplet codon in place** of K201)	ATGGGGGCGCCAAAAAAGAAGAGGAAAGTATCGAATTTGCTCACC<br>GTCCACCAAAACCTCCCAGCCCTCCCAGTCGACGCCACCTCCGAC<br>GAGGTCCGTAAGAACCTCATGGACATGTTCCGTGACCGTCAAGCC<br>TTCTCCGAGCACACCTGGAAGATGCTCCTCTCCGTCTGCCGTTCC<br>TGGGCCGCCTGGTGCAAGCTCAACAACCGTAAGTGGTTCCCAGCC<br>GAGCCAGAGGACGTCCGTGACTACCTCCTCTACCTCCAAGCCCGT<br>GGACTCGCCGTCAAGgtaagtttaaacatatatatactaactaac<br>cctgattatttaaattttcagACCATCCAACAACACCTCGGACAA<br>CTCAACATGCTCCACCGTCGTTCCGGACTCCCACGTCCATCCGAC<br>TCCAACGCCGTCTCCCTCGTCATGCGTCGTATCCGTAAGGAGAAC<br>GTCGACGCCGGAGAGCGTGCCAAGCAAGCCCTCGCCTTCGAGCGT<br>ACCGACTTCGACCAAGTCCGTTCCCTCATGGAGAACTCCGACCGT<br>TGCCAAGACATCCGTAACCTCGCCTTCCTCGGAATCGCCTACAAC<br>ACCCTCCTCCGTATCGCCGAGATCGCCCGTATCCGTGTCAAGgta<br>agtttaaacagttcggtactaactaaccatacatatttaaatttt<br>cagGACATCTCCCGTACCGACGGAGGACGTATGCTCATCCACATC<br>GGACGTACC**TAGA**ACCCTCGTCTCCACCGCCGGAGTCGAGAAGGC<br>CCTCTCCCTCGGAGTCACCAAGCTCGTCGAGCGTTGGATCTCCGT<br>CTCCGGAGTCGCCGACGACCCAAACAACTACCTCTTCTGCCGTGT<br>CCGTAAGgtaagtttaaacatgattttactaactaactaatctga<br>tttaaattttcagAACGGAGTCGCCGCCCCATCCGCCACCTCCCA<br>ACTCTCCACCCGTGCCCTCGAGGGAATCTTCGAGGCCACCCACCG<br>TCTCATCTACGGGGCCAAGGACGACTCCGGACAACGTTACCTCGC<br>CTGGTCCGGACACTCCGCCCGTGTCGGAGCCGCCCGTGACATGGC<br>CCGTGCCGGAGTCTCCATCCCAGAGATCATGCAAGCCGGAGGATG<br>GACCAACGTCAACATCGTCATGAACTACATCCGTAACCTCGACTC

	CGAGACCGGAGCCATGGTCCGTCTCCTCGAGGACGGAGACTAA
rev-Caspase-3(C271TAGA). (*C. elegans* codon optimised Caspase-3, consisting of short subunit, prodomain-containing linker and long subunit with a **TAGA quadruplet codon** in place of C271)	ATGAGCGGGGTGGATGATGATATGGCTTGTCACAAGATCCCAGTC<br>GAGGCCGACTTCCTCTACGCCTACTCCACCGCCCCAGGATACTAC<br>TCCTGGCGTAACTCCAAGGACGGATCCTGGTTCATCCAATCCCTC<br>TGCGCCATGCTCAAGCAATACGCCGACAAGCTCGAGTTCATGCAC<br>ATCCTCACCCGTGTCAACCGTAAGGTCGCCACCGAGTTCGAGTCC<br>TTCTCCTTCGACGCCACCTTCCACGCCAAGGTAAGTTTAAACATA<br>TCTATACTAACTAACCCTGATTATTTAAATTTTCAGAAGCAAATC<br>CCATGCATCGTCTCCATGCTCACCAAGGAGCTCTACTTCTACCAC<br>GACGAGGTCGACGGAATGGAGAACACCGAGAACTCCGTCGACTCC<br>AAGTCCATCAAGAACCTCGAGCCAAAGATCATCCACGGATCCGAG<br>TCCATGGACTCCGGAATCTCCCTCGACAACTCCTACAAGATGGAC<br>TACCCAGAGATGGGACTCTGCATCATCATCAACAACAAGgtaagt<br>ttaaacagttcggtactaactaaccatacatatttaaattttcag<br>AACTTCCACAAGTCCACCGGAATGACCTCCCGTTCCGGAACCGAC<br>GTCGACGCCGCCAACCTCCGTGAGACCTTCCGTAACCTCAAGTAC<br>GAGGTCCGTAACAAGAACGACCTCACCCGTGAGGAGATCGTCGAG<br>CTCATGCGTGACGTCTCCAAGGAGGACCACTCCAAGCGTTCCTCC<br>TTCGTCTGCGTCCTCCTCTCCCACGGAGAGGAGGGAATCATCTTC<br>GGAACCAACGGACCAGTCGACCTCAAGgtaagtttaaacatgatt<br>ttactaactaactaatctgatttaaattttcagAAGATCACCAAC<br>TTCTTCCGTGGAGACCGTTGCCGTTCCCTCACCGGAAAGCCAAAG<br>CTCTTCATCATCCAAGCC**TAGA**CGTGGAACCGAGCTCGACTGCGG<br>AATCGAGACCGACTAA

1. Davis, L., Radman, I., Goutou, A., Tynan, A., Baxter, K., Xi, Z., O'Shea, J.M., Chin, J.W. and Greiss, S. (2021) Precise optical control of gene expression in C elegans using improved genetic code expansion and Cre recombinase. *Elife*, **10**, e67075.
2. Greiss, S. and Chin, J.W. (2011) Expanding the genetic code of an animal. *J Am Chem Soc*, **133**, 14196-14199.
3. Radman, I., Greiss, S. and Chin, J.W. (2013) Efficient and rapid C. elegans transgenesis by bombardment and hygromycin B selection. *PLoS One*, **8**, e76019.
4. Hunt-Newbury, R., Viveiros, R., Johnsen, R., Mah, A., Anastas, D., Fang, L., Halfnight, E., Lee, D., Lin, J., Lorch, A. *et al.* (2007) High-Throughput In Vivo Analysis of Gene Expression in Caenorhabditis elegans. *PLoS Biology*, **5**, e237.
5. Nguyen, D.P., Mahesh, M., Elsässer, S.J., Hancock, S.M., Uttamapinant, C. and Chin, J.W. (2014) Genetic encoding of photocaged cysteine allows photoactivation of TEV protease in live mammalian cells. *J Am Chem Soc*, **136**, 2240-2243.
